# Struct2ML: End-to-End Prediction of Electronic Charge Densities Using 3D U‑Net

**DOI:** 10.1021/acsomega.6c07986

**Published:** 2026-08-21

**Authors:** João Victor Vilela Cassiano, Marcel Valério de Arruda, Alvaro David Torrez-Baptista, Caetano Rodrigues Miranda

**Affiliations:** 28133 Universidade de São Paulo, Instituto de Física, Rua do Matão, 1371, 05508-090 São Paulo, SP, Brazil

## Abstract

We present Struct2ML, a scalable, low-latency volumetric inference pipeline that predicts three-dimensional electronic charge densities directly from atomic configurations. By leveraging a 3D U-Net architecture, our method provides a surrogate route to approximate charge-density fields without performing a full self-consistent Kohn–Sham calculation for each queried structure, mapping multichannel structural descriptors with explicit periodic-boundary handling to volumetric charge fields. Unlike graph-based approaches that often require complex basis functions to reconstruct continuous fields, our grid-based representation maintains a computational complexity of O(N) , offering a natural interface for large-scale periodic and molecular systems optimized for real-time analysis. Key contributions include (i) scalable infrastructure, a high-throughput disk-to-GPU streaming dataloader optimized for terabyte-scale HDF5 archives, ensuring efficient training on massive data sets; (ii) robust training protocol, a reproducible workflow featuring automated voxel normalization, physics-informed multiobjective loss functions, and rigorous checkpointing; and (iii) multi-domain validation, a comprehensive evaluation suite providing both voxel-wise quantitative metrics and qualitative spatial analysis tailored for charge-density comparison. By substantially reducing the inference time to the millisecond scale (1–10 ms), this framework enables real-time charge-density exploration that reproduces key spatial and topological features of reference density functional theory (DFT) densities with data set-dependent accuracy, serving as a high-throughput utility complementary to equivariant models that primarily target static, maximal accuracy.

## Graphical abstract

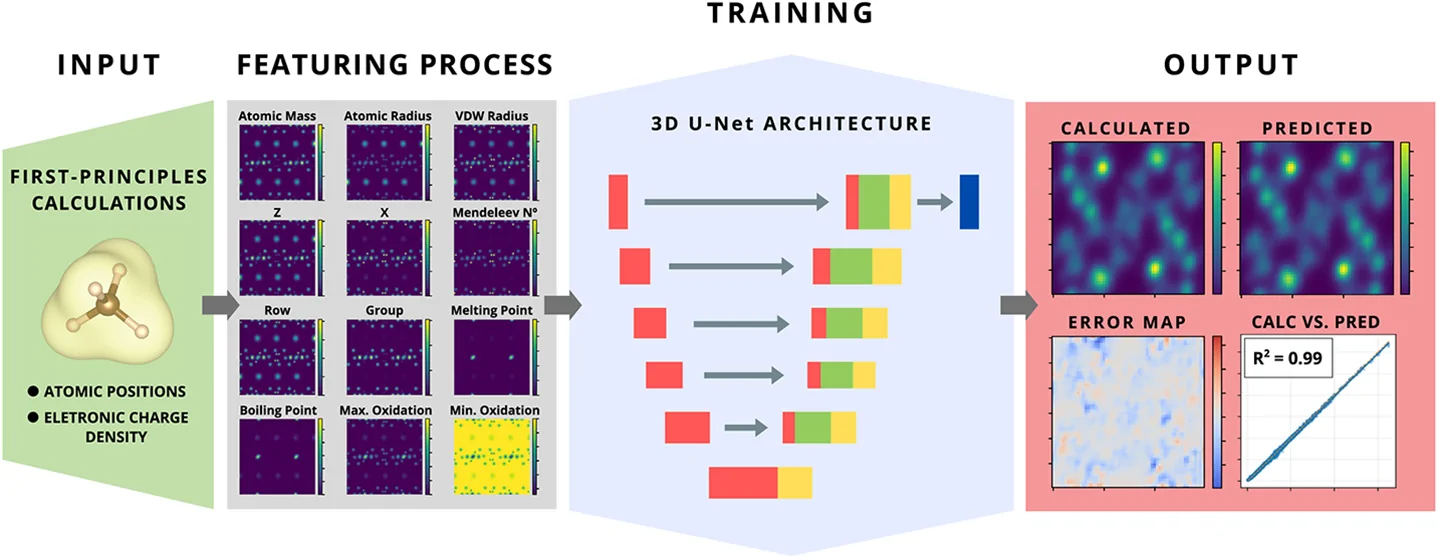

## 1 Introduction

Density functional theory (DFT) has become the standard framework for describing the electronic structure of molecules and solids by recasting the many-electron problem in terms of the electron density ρ­(**r**).^1–4^ In practical materials modeling, DFT is most commonly solved using the Kohn–Sham formulation and implemented in plane wave or localized basis set codes such as VASP,^5,6^ QuantumEspresso,^7^ SIESTA,^8^ ABINIT,^9^ among others. These calculations provide access not only to total energies and forces but also to real-space charge-density fields used in bonding analysis, electrostatics, and materials characterization.^5,6,10^ Despite its broad success, generating three-dimensional charge densities at scale remains computationally demanding, particularly when exploring large compositional spaces, sampling finite-temperature configurations, or constructing high-throughput data sets for screening and discovery.

Although DFT is the standard, scalability limitations have motivated the use of machine learning (ML), which has emerged as a central methodology in computational materials science, enabling surrogate models that accelerate or augment first-principles workflows across tasks such as property prediction, interatomic potentials, and structure–property mapping.^11–13^ Beyond materials modeling, ML has permeated chemistry and neighboring disciplines at large, being applied to correlate and reconcile results obtained at different levels of electronic-structure theory,^14^ to design functional materials and chemical sensors,^15,16^ and to accelerate biomedical discovery through the virtual screening of candidate inhibitors.^17^ This breadth, spanning the properties and reactivity of small organic and inorganic molecules, the design of polymers and composites, and biomedically oriented applications, illustrates how data-driven models have become a general-purpose tool across chemistry, materials science, and related fields.^18^ The growing availability of open materials databases and programmatic toolchains has further catalyzed data-driven approaches, with the Materials Project (MP) and the Pymatgen ecosystem providing standardized access to crystal structures and derived quantities that support reproducible model development.^19,20^ Within this broader landscape, learning electronic-structure ingredients directly from atomic configurations is especially attractive because it targets a key intermediate representation of DFT, the electron density ρ­(**r**), and thereby enables downstream electronic-structure observables computed from ρ­(**r**) without running an explicit self-consistent field cycle.

Recent progress has shown that ML models can approximate electron densities and related electronic fields while partially bypassing the iterative self-consistency of Kohn–Sham DFT. Examples include direct learning strategies that emulate the density map as a function of atomic environments, symmetry-preserving neural architectures, and transferable density models across chemical systems.^21–23^ For crystalline materials, graph-based and local-environment models have demonstrated that charge-density distributions can be predicted with physically meaningful locality constraints and improved generalization across structure classes.^24–26^ Nevertheless, accurately learning *full* volumetric densities in three dimensions remains challenging due to the high dimensionality of the output, the need for the robust handling of periodic boundary conditions (PBCs), and the practical constraints imposed by large-scale storage, preprocessing, and training throughput.

While early work by Sinitskiy and Pande^27^ pioneered DNN-based voxel/CNN approaches for charge density prediction, the field has recently seen a shift toward graph-based models. DeepDFT^28^ formulates density prediction via message passing with query points, and more recent approaches further incorporate explicit geometric equivariance.^26,29^ In particular, ChargE3Net^29^ represents a strong state of the art in symmetry handling and accuracy for density prediction. However, these graph-based methods typically prioritize accuracy over throughput, and their end-to-end latency can depend strongly on the implementation, reconstruction strategy, and available hardware.

Many graph-based approaches require an additional reconstruction or query-point evaluation step (e.g., interpolation to a mesh or expansion in atom-centered basis functions) to obtain a dense three-dimensional field from discrete node representations. In contrast, a voxel architecture based on the U-Net predicts the target directly in its native real-space grid representation.^30–32^ The U-Net is efficient at capturing features across multiple length scales, which is essential for electronic-structure fields that exhibit both sharp nuclear cusps and smooth, long-range variations in bonding regions.

The native grid prediction is particularly attractive for electronic-structure learning because many downstream quantities are naturally represented and manipulated on uniform real-space meshes (densities, electrostatic/Hartree and exchange–correlation potentials, and other volumetric observables): grid outputs can be postprocessed with standard numerical operators (integration, finite differences, fast Fourier transform (FFT)-based Poisson solvers), periodic boundary conditions can be applied straightforwardly, and existing workflows that consume volumetric data can be used without an extra mesh/basis conversion step. Once the unit cell or molecule is aligned in a standard reference frame, the 3D U-Net also leverages highly optimized convolutions to generate millions of grid values quickly, enabling high-throughput screening and on-the-fly electronic-structure analysis.

In this work, we introduce *Struct2ML*, an end-to-end engineering framework designed to reproduce key spatial features of first-principles 3D volumetric charge densities directly from atomistic structure inputs, including data retrieved from the Materials Project.^19,20^ Our approach represents each structure as a multichannel 3D tensor encoding physically motivated atomic descriptors on a fixed voxel lattice, training a volumetric convolutional model based on the U-Net family to learn multiscale spatial correlations.^30–32^ To encourage both voxelwise fidelity and global structural agreement, we employ overlap-aware losses rooted in the Dice coefficient alongside complementary error terms and similarity measures.^32–34^ Designed for data set-scale learning, the pipeline utilizes HDF5-backed storage and streaming to support terabyte-class training archives,^35^ relying on standard scientific-computing primitives for consistent resampling and preprocessing.^36^ Rather than focusing on continuous structural invariant mappings, this framework addresses a practical engineering gap: the realization of a high-throughput inference pipeline yielding stable predictions within a 1–10 ms regime on standard GPU hardware. This low-latency execution is tailored to support interactive downstream workflows, such as on-the-fly visualization or real-time spatial analysis, offering a scalable engineering contribution for large-scale systems where the immediate observation of density rearrangements can accelerate materials exploration.

To clearly situate this approach within the broader landscape of machine learning for electronic structure emulation, Table 1 contrasts the fundamental design choices of *Struct2ML* with those of representative paradigms in the literature. This selection spans early deep neural networks for molecular fields,^27^ crystalline descriptor-based local graph networks,^24^ neural message-passing implementations,^28^ and state-of-the-art higher-order equivariant graph architectures.^29^ For an extended, comprehensive literature-based analysis encompassing a broader set of framework variants and target sampling configurations, the reader is referred to Section 3 of the Supporting Information (see Table S3).

**Table 1:** Methodological Comparison of Representative Machine-Learning Strategies for Electron-Density Prediction^a^

method	input and density representation	symmetry and inductive bias	primary design focus
Sinitskiy and Pande^27^	molecular spatial fields mapped onto a volumetric grid.	no explicit *E*(3)-equivariance implemented.	accelerating molecular field evaluations.
Gong et al. GCN^24^	local-environment descriptors mapped to graph nodes.	localized geometric descriptors; not fully equivariant.	crystalline material structural mapping.
DeepDFT^26,28^	graph model evaluated over discrete query points.	message-passing localized interactions.	accuracy-oriented point-wise field learning.
ChargE3Net^29^	equivariant field models on atom-centered spheres.	explicit higher-order *E*(3)-equivariant features.	rigorous physical symmetry constraints.
Struct2ML	fixed 3D volumetric grid with 12-channel descriptors.	explicit periodic boundaries via modulo mapping.	low-latency processing and data streaming.

a Because training datasets, coordinate representations, and evaluation protocols differ across the original studies, this overview outlines qualitative architectural positioning rather than a direct performance ranking.

To complement the qualitative positioning in Table 1, Figure 1 places these representative works on a common timeline and reports, for each one, the originally published accuracy figure alongside the error obtained when Struct2ML predictions are evaluated under that paper’s own comparable-metric protocol (CO_2_, CH_4_, and Materials Project targets). Because normalization conventions, target definitions, and evaluation domains differ substantially across studies, these comparable-metric values should be interpreted as an approximate, protocol-matched cross-check rather than a like-for-like benchmark; nonetheless, the consistent pattern across studies indicates that Struct2ML operates within a comparable accuracy regime to prior work.

**Figure 1:**
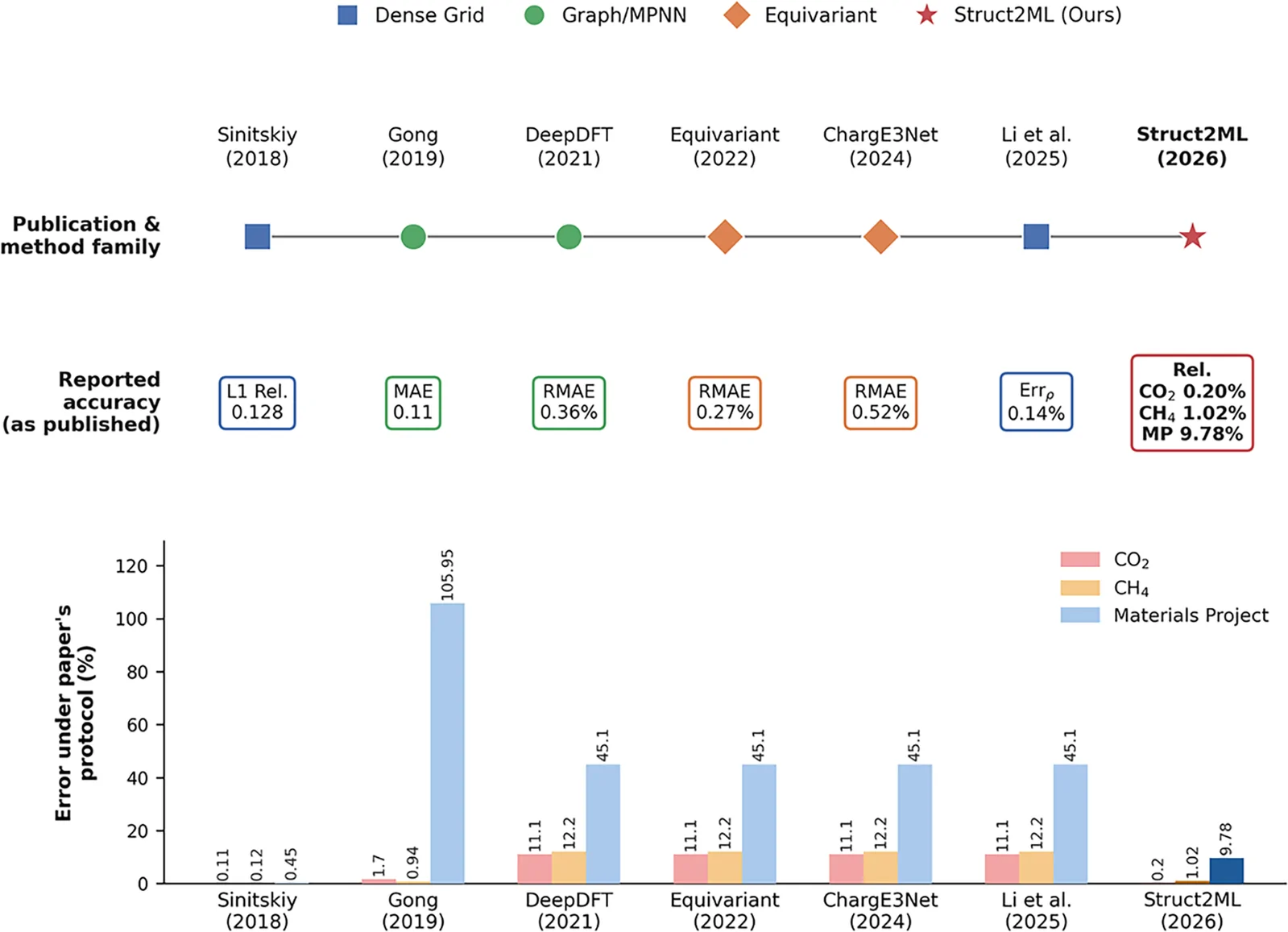
Timeline comparison of representative electron-density prediction methods against Struct2ML. From top to bottom: publication year and method family (Dense Grid, Graph/MPNN, Equivariant, or Struct2ML); the accuracy metric as originally reported in each study; and the error obtained when Struct2ML predictions are re-evaluated under each paper’s own comparable-metric protocol for CO_2_, CH_4_, and Materials Project (MP) targets.

Rather than evaluating these methodologies through a direct numerical ranking, which remains fundamentally constrained by disparate target normalization protocols, data set splitting choices, and resolution scales, our focus centers on architectural design trade-offs. Symmetry-aware implementations, such as the equivariant graph neural network (GNN) configuration seen in ChargE3Net,^29^ achieve high spatial accuracy by embedding rigorous *E*(3)-equivariance constraints natively into tensor-product message-passing loops. This physical prioritization inherently introduces nontrivial scaling relationships with respect to coordinate tracking, system size, and neighbor list graph construction.

In contrast, *Struct2ML* operates on a complementary engineering objective, trading strict continuous rotational equivariance for an enhanced computational throughput and data streaming efficiency. By utilizing standard voxelized convolutions over the predefined fixed grid, the framework avoids dynamic runtime graph reconstruction and localized coordinate query sampling, representing a practical compromise that optimizes scalable data handling. Consequently, this supports low-latency fixed-grid inference in workflows where direct volumetric access is prioritized, such as downstream streaming data environments, high-throughput screening, or interactive structural exploration.

## 2 Methodology

To train a 3D U-Net capable of predicting electronic charge density fields from atomic structures, all configurations, whether obtained from the Materials Project API^19,20^ or generated locally through first-principles calculations, must be converted into a consistent volumetric representation. Each system is transformed into a fixed-size multichannel 3D grid that encodes atomic positions, periodicity, and element-specific physical descriptors, while the corresponding charge-density field is resampled onto the same lattice. These standardized representations enable the 3D U-Net to learn spatial correlations and multiscale electronic patterns directly from voxelized data. Additional analyses regarding the optimization trajectory leading to the final 12-channel representation, as well as explainability investigations of the learned latent representations and attribution patterns, are provided in the Supporting Information.

Training is performed using a weighted multiterm loss function that combines several complementary components, including pointwise mean absolute error (MAE), Soft Dice loss, and additional regularization terms, each contributing to different aspects of model performance. This composite objective simultaneously promotes voxelwise numerical accuracy, volumetric overlap fidelity, and smoothness or stability in the predicted charge density fields, promoting spatially coherent predictions and improved agreement with reference density fields. The complete preprocessing and learning workflow is summarized in Figure 2. The subsections below detail the complete sampling pipeline, including structure voxelization, Gaussian smoothing of atomic environments, grid resampling, and processing of data from both external databases and local first-principles simulations.

**Figure 2:**
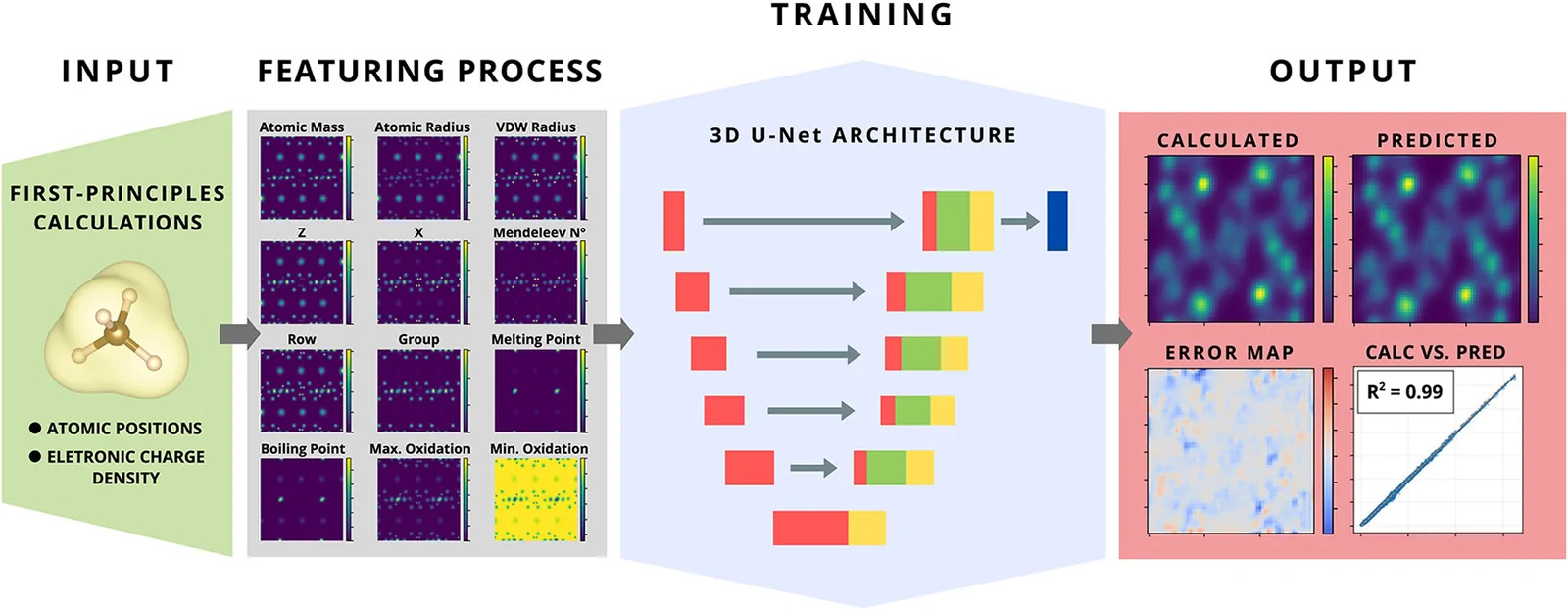
Overview of the data preprocessing and learning pipeline. Structures are converted into multichannel 3D voxel grids encoding atomic environments and physical descriptors. Corresponding VASP charge-density fields are resampled onto the same lattice. These paired volumes are then used to train the 3D U-Net for direct density prediction.

### 2.1 Sampling of Atomic Structures into 3D Multi-Channel Grids

To convert each atomic structure into a fixed-size volumetric representation suitable for 3D convolutional neural networks, we implemented a unified voxelization pipeline that encodes atomic positions, element-specific physical descriptors, Gaussian-smoothed atomic fields, and charge density maps. The method is applied consistently across all data sets, including VASP-generated simulations and Materials Project entries.

#### 2.1.1 Materials Project Retrieval and Caching

Structures containing total charge-density data were obtained from the Materials Project (MP) repository via the MPRester API, using a composition filter to restrict the data set (e.g., to C-containing systems). For each material with ID *m*, both the Structure object and the charge-density array were downloaded and cached locally as <mpid>.npy files. The pipeline supports automatic resumption: previously processed structures are skipped when rebuilding the NPZ/HDF5 data sets.

#### 2.1.2 Processing of Local First-Principles Calculations

In addition to Materials Project data, we also build a robust data set by performing first-principles calculations using VASP^5,6,10^ to analyze the electronic properties of methane (CH_4_). We employ a multistep computational approach. This involves selecting representative snapshots from ab initio molecular dynamics (AIMD) trajectories and subsequently performing high-precision static calculations. A sampling of CH_4_ configurations was performed by using AIMD simulations in the gas phase. To ensure adequate thermal exploration of the phase space, trajectories were generated in the canonical ensemble (NVT) at 300 K using a time step of 1 fs. The simulation totaled a time scale of 20 ps, providing a statistically relevant database and diverse molecular configurations for subsequent charge density analysis.

To compose a comprehensive database, we selected representative snapshots from our AIMD simulation. This sampling strategy allows for the capture of diverse local environments and vibrational configurations inherent to the CH_4_ system at finite temperatures. For each extracted snapshot, the electronic charge density was calculated by using the VASP code. To maintain numerical consistency in all CH_4_ snapshots, we used the accurate precision to automatically set the scales of the fast Fourier transform (FFT) grids to avoid wrap-around errors and ensure that the kinetic energy cutoff of 850 eV is fully utilized without interpolation artifacts.

By combining a tight electronic convergence (10^–6^ eV) with high-precision grid settings, the resulting electronic charge density provides a robust foundation for subsequent analysis, such as input for machine learning models. It is important to note that our convergence criteria are significantly more stringent than those typically found in high-throughput databases, such as the MP. While MP parameters are optimized for bulk crystalline materials, the simulation of gas-phase molecular snapshots from AIMD demands higher accuracy. In these systems, the electronic density is highly sensitive to small structural fluctuations; therefore, adopting more rigorous convergence thresholds is imperative to ensure numerical stability and accurate mapping of the electronic distribution.

Each simulation produced a directory of the form snapshot-XXXX containing an atomic structure (POSCAR file) and total charge density (CHGCAR file). All snapshots were automatically discovered via directory pattern matching, and a resume mechanism ensured that previously processed snapshots were skipped when rebuilding the data set. For each snapshot, the atomic structure was loaded as a Pymatgen Structure, and the total charge-density array ρ_VASP_ was extracted from the CHGCAR file. The atomic structure was converted into a multichannel voxel grid **G** using the same pipeline applied to Materials Project structures (fractional coordinate mapping, property-channel assignment, species-wise Gaussian spreading). The corresponding charge-density grid was then resampled to the target resolution (*N*_*x*_, *N*_*y*_, *N*_*z*_) using trilinear interpolation,^36^ eq 1

1 $$\rho_{\mathrm{resampled}}=\mathrm{zoom}\left(\rho_{\mathrm{VASP}},\mathrm{scale}=\left(N_{x}/n_{x},N_{y}/n_{y},N_{z}/n_{z}\right),\mathrm{order}=1\right)$$

where (*n*_*x*_, *n*_*y*_, *n*_*z*_) is the native VASP grid shape.

The choice of a uniform grid resolution for both input descriptors and target densities ensures a direct spatial alignment and minimizes interpolation artifacts. While multiscale architectures or denser target resampling are potential avenues for future refinement, our current uniform approach prioritizes high-throughput efficiency for low-latency, interactive analysis, maintaining an O(N) complexity.

Both the structure grids and charge-density grids were stored in HDF5 files, with snapshot names used as data set keys. Batching and periodic flushing to disk ensured scalability for large simulations,^37^ while errors encountered in individual snapshots were logged without interrupting the full processing pipeline. This provided a robust and fully automated method for converting large-scale VASP simulations into uniform 3D machine-learning-ready data sets.

#### 2.1.3 Multichannel Atomic Property Grid

Each structure with fractional coordinates **f** = (*f*_*x*_, *f*_*y*_, *f*_*z*_) is discretized onto an (*N*_*x*_, *N*_*y*_, *N*_*z*_) voxel grid by mapping

2 $$\left(i,j,k\right)=\left(\lfloor f_{x}N_{x}\rfloor \;\mathrm{mod}N_{x},\lfloor f_{y}N_{y}\rfloor \;\mathrm{mod}N_{y},\lfloor f_{z}N_{z}\rfloor \;\mathrm{mod}N_{z}\right)$$

which automatically enforces periodic boundary conditions (PBCs).

#### 2.1.4 Symmetry Considerations

The present representation and architecture exploit a subset of the symmetries of the underlying electronic-structure problem. First, periodic boundary conditions are explicitly enforced at the *representation* level through the modulo mapping in eq 2, which ensures that atoms translated by integer lattice vectors are mapped to equivalent voxel indices. Second, because the predictor is based on 3D convolutions, it is (approximately) translation-equivariant *within the interior of the grid*: shifting the input structure tensor by an integer number of voxels tends to shift the predicted density by the same amount. However, the model is *not* guaranteed to be strictly equivariant to global translations when boundary effects (finite grid extent, padding choices, and interpolation/resampling of ρ) are considered.

In contrast, neither the voxel embedding nor the standard 3D U-Net is intrinsically equivariant to general 3D rotations or to crystal space group operations. Rotations and point/space group symmetries are therefore only learned implicitly from the data distribution unless explicitly promoted via symmetry-aware augmentation or equivariance-regularizing objectives. In the experiments below, we quantify these limitations and assess the effect of symmetry-aware augmentation on generalization.

Instead of storing only atom counts, each atom contributes a 12-dimensional descriptor vector (eq 3 and Figure 3). The channels include core physical attributes (e.g., nuclear charge and length-scale proxies) together with additional tabulated chemical properties used as *auxiliary embeddings* to enrich the element identity. In particular, quantities such as melting and boiling points are not treated as direct physical observables of the specific atomic configuration but as fixed element-level indicators that can improve separability across species.

**Figure 3:**
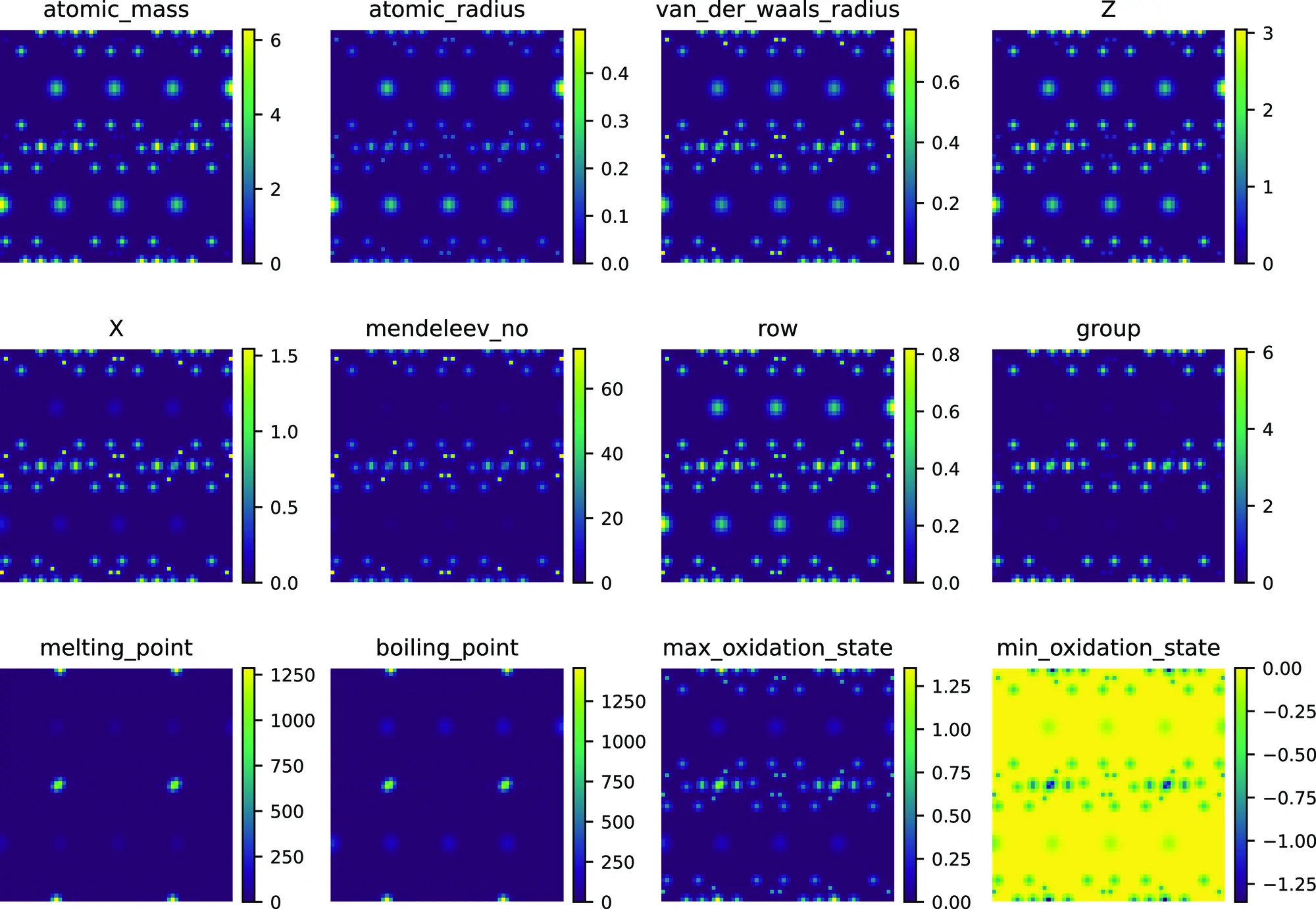
Twelve-channel voxel representation of atomic descriptors for a representative Materials Project structure (mp-1001112). Each panel shows the *z*-integrated projection of one normalized descriptor channel (e.g., atomic mass, radii, *Z*, electronegativity, Mendeleev number, period/group, tabulated melting/boiling points used as auxiliary element embeddings, and oxidation-state bounds) after species-wise Gaussian spreading and resampling onto a common 60^3^ grid.

The rationale behind the feature selection and the optimization trajectory leading to the final 12-channel representation are described in the Supporting Information. Our ablation analysis (Supporting Information Section 2) indicates that the full 12-channel configuration improves robustness and reduces variance in prediction error across chemically diverse systems compared to reduced-feature models.

3 $$p=\left[\begin{array}{llll} \mathrm{atomic}\;\mathrm{mass} & \mathrm{atomic}\;\mathrm{radius} & \mathrm{vdW}\;\mathrm{radius} & Z\\ \mathrm{electronegativity} & \mathrm{Mendeleev}\;\mathrm{no.} & \mathrm{period} & \mathrm{group}\\ T_{\mathrm{melt}} & T_{\mathrm{boil}} & {\mathrm{ox}}_{\max} & {\mathrm{ox}}_{\min} \end{array}\right]$$

Each property defines one channel of the feature tensor, resulting in the following representation

4 $$G\in R^{C\times N_{x}\times N_{y}\times N_{z}},C=12$$

For every atom assigned to voxel (*i*, *j*, *k*), the channel values are accumulated: *G*_*c*,*i*,*j*,*k*_ + = *p*_*c*_.

#### 2.1.5 Species-wise Gaussian Spreading

To transform the sparse impulse lattice into a smooth, physically meaningful field, each atomic species *s* undergoes an element-dependent Gaussian spreading. The standard deviation σ*_s_* is derived from the atomic radius *r_s_*

5 $$\sigma_{s}=\min\left(\sqrt{r_{s}},10\right)$$

where the upper bound ensures numerical stability for heavy elements. Each species-specific channel is then convolved with a 3D Gaussian kernel, $N\left(0,\sigma_{s}^{2}\right)$ , to produce a continuous feature map

6 $${\mathrm{G̃}}^{\left(s\right)}c=G^{\left(s\right)}c\times N\left(0,\sigma_{s}^{2}\right)$$

The final voxel grid *G*_*c*_ is obtained by summing the contributions from all species

7 $$G_{c}=\sum_{s}{\mathrm{G̃}}_{c}^{\left(s\right)}$$

This procedure generates continuous spatial fields that reflect local atomic environments with a smooth decay in influence away from each nucleus, providing a robust input representation for the 3D U-Net.

#### 2.1.6 Charge-Density Interpolation

Materials Project and VASP charge-density grids, which vary in resolution, are resampled to the target lattice via trilinear interpolation

8 $$\rho_{\mathrm{resampled}}\left(x\right)=\mathrm{zoom}\left(\rho_{\mathrm{raw}},\mathrm{scale},\mathrm{order}=1\right)$$

where the scale factors match the desired (*M*_*x*_, *M*_*y*_, *M*_*z*_) grid dimensions. This ensures a uniform representation across all materials.

#### 2.1.7 3D Grid Resampling

After Gaussian spreading, the multichannel atomic grid is likewise standardized

9 $$Ĝ=\mathrm{zoom}\left(G,\mathrm{scale}=\left(M_{x}/N_{x},M_{y}/N_{y},M_{z}/N_{z}\right),\mathrm{order}=1\right)$$

This guarantees that all structures, regardless of the unit-cell shape or size, are encoded on a common voxel lattice.

#### 2.1.8 Implementation

The pipeline is implemented in two core routines. The first constructs the smoothed multichannel grid:

def load_3d_grid_multichannel_2­(... ):

...

The second performs trilinear resampling:

def resample_3d­(...):

...

Together, they provide a consistent, physics-informed, resolution-controlled 3D representation of atomic structures and their associated charge densities, enabling efficient learning with 3D U-net architectures.

### 2.2 Normalization of Inputs, Targets, and Loss Components

To ensure stable optimization and consistent learning behavior across heterogeneous systems, all volumetric channels and charge-density fields are normalized prior to training. Without normalization, the large dynamic range inherent to both atomic descriptors and raw charge-density values can cause the 3D U–Net to overfit high-magnitude regions or suffer from vanishing gradients in low-density regions. Our normalization strategy operates at three complementary levels: (i) channel-wise normalization of the multichannel atomic grid, (ii) global or per-sample normalization of charge-density targets, and (iii) internal normalization of all loss components to enforce scale-invariant optimization.

#### 2.2.1 Normalization of Atomic Descriptor Channels

Each of the *C* = 12 physical descriptor channels in the voxelized structure grid spans distinct numerical ranges (e.g., atomic mass versus oxidation state). To prevent any descriptor from dominating the feature space, each channel *c* is normalized independently using a min–max transformation^38,39^ computed over the full training corpus following the eq 10

10 $$G_{c,i,j,k}^{\mathrm{norm}}=\frac{G_{c,i,j,k}-\min\left(G_{c}\right)}{\max\left(G_{c}\right)-\min\left(G_{c}\right)+\epsilon },G_{c,i,j,k}^{\mathrm{norm}}\in \left[0,1\right]$$

This produces a unified, dimensionless representation where all descriptor channels contribute comparably to the early convolutional layers of the 3D U–Net.

#### 2.2.2 Normalization of Charge-Density Targets

The raw charge-density values ρ_raw_ collected from either CHGCAR file or Materials Project data exhibit substantial variation caused by differences in unit-cell volume, atomic composition, and electron count. Charge-density volumes are normalized via a global scaling factor or a per-sample approach (eq 11) to ensure that the network predicts values within a consistent range

11 $$\rho_{\mathrm{norm}}\left(x\right)=\frac{\rho_{\mathrm{raw}}\left(x\right)}{\rho_{\max}+\epsilon },\rho_{\mathrm{norm}}\in \left[0,1\right]$$

For data sets in which electron-density magnitudes vary significantly between systems, a per-sample maximum ρ_max_ is used. For homogeneous data sets (e.g., fixed composition or constant electron count), a corpus-wide ρ_max_ is applied. This normalization is crucial for training the model with a sigmoid output layer constrained to [0, 1].

Unless explicitly stated otherwise, all evaluation metrics reported in Section 3 are computed on the *denormalized* charge density (eq 12), i.e., after inverting the scaling above for both prediction and reference

12 $$\rho_{\mathrm{raw}}\left(x\right)\approx \rho_{\mathrm{norm}}\left(x\right)\left(\rho_{\max}+\epsilon \right)$$

Consequently, MAE and root-mean-squared error (RMSE) are reported in the same physical units as the underlying VASP/Materials Project charge-density grids (*e*/Å^3^), while the relative error and *R* ^2^ are dimensionless. This convention avoids reporting MAE/RMSE in arbitrary [0, 1] units that would depend on the choice of ρ_max_.

#### 2.2.3 Normalization Inside the Loss Function

All error terms entering the combined loss are internally normalized to enforce scale invariance: (i) the mean squared error (MSE) term is divided by the variance of the ground-truth volume,^40,41^ (ii) relative L1 explicitly divides by ∥*y*∥_1_, (iii) projection MSE is normalized by the square of the mean axial projection, and (iv) dice loss is clamped to prevent numerical divergence.

These internal normalizations ensure that no single component disproportionately influences the optimization because of scale differences.

#### 2.2.4 Normalization of Learnable Loss Weights

Finally, after weighting each loss component by its learnable weight *w*_*i*_, the combined loss applies a normalization factor based on the magnitude of all weights, following eq 13

13 $$L_{c\mathrm{ombined}}=\frac{\sqrt{\sum_{i}{\left(w_{i}L_{i}\right)}^{2}}}{\sqrt{\sum_{i}w_{i}^{2}}+\epsilon }$$

which prevents pathological behavior (e.g., exploding weights) and ensures that the overall loss remains comparable across training epochs.

#### 2.2.5 Impact on Model Stability

Together, these normalization procedures lead to substantially improved convergence stability across heterogeneous materials; enhanced gradient balance between low- and high-density regions; reduced sensitivity to outliers in atomic descriptors or charge-density magnitudes; consistent activation statistics throughout the 3D U–Net.^42–44^

This comprehensive normalization strategy is essential for effective large-scale training of thousands of structurally diverse systems. By enabling the network to learn physically meaningful charge density mappings without being biased by differences in descriptor scaling or density amplitude, we inherently mitigate predictive uncertainties across heterogeneous samples. The impact of this controlled uncertainty is directly managed and quantified by our multiobjective optimization approach, specifically the combined loss function detailed in Section 2.4.

### 2.3 3D U-Net Architecture

We implemented a 3D U-Net architecture^30,31^ tailored for volumetric charge-density prediction and auxiliary reconstruction. The network follows a symmetric encoder–decoder topology with skip connections, enabling simultaneous extraction of multiscale spatial features and preservation of fine-grained structural information. The model receives a 3D volume as input (12-channel unless otherwise specified) and produces two outputs: (i) a normalized prediction of the target 3D density map and (ii) a reconstructed version of the input volume used for the autoencoder-based loss component.

#### 2.3.1 Encoder

The encoder path comprises two resolution levels, each containing two consecutive 3D convolutional blocks (3 × 3 × 3 kernels, batch normalization, and ReLU activation). Downsampling is performed via 2 × 2 × 2 max-pooling. Specifically, the first level (Encoder 1) applies Conv­(*C*_in_ → *F*) followed by Conv­(*F* → *F*), while the second level (Encoder 2) increases the feature depth using Conv­(*F* → 2*F*) and Conv­(2*F* → 2*F*).

#### 2.3.2 Bottleneck

At the lowest resolution level, the bottleneck block extracts high-level volumetric features using two convolutional layers, Conv­(2*F* → 4*F*) and Conv­(4*F* → 4*F*). This stage is designed to capture the global contextual information critical for accurate volumetric prediction across the entire domain. The interpretability of these latent representations was further investigated through complementary gradient-based and SHAP-based attribution analyses (Supporting Information).

#### 2.3.3 Decoder

The recovery path mirrors the encoder through two up-sampling levels, restoring the feature maps to their original spatial resolution. Skip connections are incorporated by concatenating the up-sampled representations with the corresponding encoder features to preserve fine spatial details. Specifically, in the first stage (Decoder 2), the bottleneck output is up-sampled via UpConv­(4*F* → 2*F*), concatenated with Encoder 2 features, and processed by two convolutional layers: Conv­(4*F* → 2*F*) and Conv­(2*F* → 2*F*). In the final stage (Decoder 1), the representation is further up-sampled using UpConv­(2*F* → *F*), concatenated with Encoder 1 features, and passed through Conv­(2*F* → *F*) and Conv­(*F* → *F*) blocks.

#### 2.3.4 Output Heads

Two parallel prediction heads are attached to the final decoder feature map to perform distinct tasks. The first head produces the predicted density volume via a 1 × 1 × 1 convolution, *ŷ* = σ­(Conv­(*F* → *C*_out_)), where σ denotes the sigmoid activation function to ensure voxelwise outputs within the [0, 1] range. Simultaneously, a reconstruction head (autoencoder) applies a 1 × 1 × 1 convolution to reconstruct the input volume, *x̂* = Conv­(*F* → *C*_in_), which is utilized in the structural similarity reconstruction loss to regularize the latent representations.

#### 2.3.5 Forward Pass

For an input 3D volume *x*, the encoder generates feature maps (Enc1, Enc2), which are compressed into a bottleneck representation before being up-sampled through the decoder’s hierarchical skip connections to yield the final representation dec1. The model simultaneously outputs the density prediction and the reconstruction, (*ŷ*, *x̂*) = UNet3D­(*x*). This dual-output architecture enables the network to achieve voxelwise accuracy for the charge density while enforcing structural constraints through the self-supervised reconstruction pathway.

### 2.4 Combined Loss Function

To optimize the 3D U-Net model, we employed a multiobjective loss function designed to jointly constrain voxelwise accuracy, structural similarity, relative error, and projection consistency. The proposed loss, termed the *Combined Loss*, integrates six complementary components: mean squared error (MSE), root-mean-squared error (RMSE), Soft Dice loss,^32,33^ relative L1 error, projection MSE, and an autoencoder-based structural similarity term. Each component is associated with a learnable positive weight, enabling the network to dynamically balance the heterogeneous error sources during training.

#### 2.4.1 Base Loss Components

Let *y* and *ŷ* denote the ground-truth and predicted charge densities, respectively. The objective function incorporates several base components to ensure physical and structural accuracy. Voxelwise deviations are first captured by a normalized Mean Squared Error, $L_{\mathrm{MSE}}=\mathrm{clamp}\left(∥ŷ-y{∥}_{2}^{2}/\left(\mathrm{var}\left(y\right)+\epsilon \right),0,1\right)$ , which ensures scale invariance relative to the target volume variance, and its corresponding root, $L_{\mathrm{RMSE}}=\sqrt{L_{\mathrm{MSE}}}$ , to provide an interpretable error scale in physical units while mitigating the dominance of extreme nuclear-density peaks.

To enforce proportional error minimization across samples with large dynamic ranges, we include a relative L1 error, defined by following expression

14 $$L_{r\mathrm{el}}=\mathrm{clamp}\left(∥ŷ-y{∥}_{1}/\left(∥y{∥}_{1}+\epsilon \right),0,1\right)$$

Spatial and morphological agreement is quantified via a differentiable Soft Dice formulation

15 $$L_{d\mathrm{ice}}=1-\mathrm{clamp}\left(\frac{2\sum_{i}{ŷ}_{i}y_{i}+\epsilon }{\sum_{i}{ŷ}_{i}^{2}+\sum_{i}y_{i}^{2}+\epsilon },0,1\right)$$

this term emphasizes structural overlap rather than voxelwise magnitude. Discrepancies in axial charge aggregation are further penalized by a projection-based MSE, $L_{p\mathrm{roj}}=\mathrm{clamp}\left(\mathrm{MSE}\left(\sum_{z}ŷ,\sum_{z}y\right)/\left(\mathrm{mean}{\left(\sum_{z}y\right)}^{2}+\epsilon \right),0,1\right)$ . Finally, when the auxiliary autoencoder branch is active, its reconstruction *x̂* is compared to the input *x* via a 3D structural similarity index (SSIM) module, $L_{\mathrm{AE}}=1-\mathrm{SSIM}\left(\mathrm{x̂},x\right)$ , to regularize the latent representations.

#### 2.4.2 Learnable Weights and Normalization

To equilibrate the multiobjective optimization, each loss component $L_{i}$ is associated with a learnable log-weight, log *w*_*i*_, where positivity is ensured via the transformation *w*_*i*_ = exp­(log *w*_*i*_) followed by clamping to prevent numerical divergence. Defining the loss vector $L=\left(L_{\mathrm{MSE}},L_{\mathrm{RMSE}},L_{d\mathrm{ice}},L_{r\mathrm{el}},L_{p\mathrm{roj}},L_{\mathrm{AE}}\right)$ and its corresponding weight vector **w**, the combined objective is computed using an *L*_2_ aggregation normalized by the total weight magnitude

16 $$L_{c\mathrm{ombined}}=\frac{\sqrt{\sum_{i}{\left(w_{i}L_{i}\right)}^{2}}}{\sqrt{\sum_{i}w_{i}^{2}}+\epsilon }$$

This adaptive formulation prevents any single term from dominating the gradient flow while allowing the network to prioritize specific physical or structural constraints during the different training stages. The routine logs each individual component and its learned weight to facilitate monitoring, interpretation, and subsequent ablation analysis. The final values of the learnable parameters indicated a prioritization of structural and morphological terms (Soft Dice and SSIM) during the initial epochs, followed by a stabilization of the voxel-wise error terms. This dynamic balancing prevents gradient dominance by any single loss component, helping the model capture both high-density peaks and low-density bonding regions with comparable fidelity.

### 2.5 Engineering for High-Throughput Inference

To support low-latency, interactive, and high-throughput use cases, the Struct2ML pipeline incorporates engineering optimizations designed to minimize end-to-end latency while maintaining physical consistency. Unlike typical 3D-CNN implementations, our framework utilizes a streamlined HDF5-backed data-loading protocol that bypasses standard disk I/O bottlenecks during both training and evaluation. The computational complexity of the 3D U-Net scales linearly with the number of grid voxels, $O\left(N_{\mathrm{voxels}}\right)$ , ensuring predictable latency across diverse atomic configurations.

A central contribution is the integration of the normalization formalism described in Section 2.4 with a GPU-accelerated voxelization step. This reduces preprocessing of atomic configurations to the millisecond scale. Together, these optimizations enable an end-to-end inference budget of 1–10 ms per structure, approximately 2 orders of magnitude faster than current *E*(3)-equivariant graph-based architectures that additionally require mesh reconstruction (see Table S1 and Section 3 of the Supporting Information for quantitative and architectural comparisons).

### 2.6 Symmetry-Aware Augmentation

Because standard voxel CNNs are not intrinsically equivariant to 3D rotations or space group operations, we consider symmetry-aware data augmentation to promote robustness under symmetry transformations.

#### 2.6.1 Rotation Augmentation for Molecules

For molecular data sets (CH_4_, CO_2_), during training, we apply random rigid rotations *R* ∈ SO(3) to both the input structure tensor **G** and the target density ρ­(**r**) using trilinear interpolation on the voxel grid. This promotes approximate rotational invariance of the learned mapping while preserving the end-to-end volumetric formulation.

#### 2.6.2 Symmetry-Allowed Augmentation for Crystalline Systems

For these systems, we restrict augmentations to symmetry operations that keep the structure invariant up to lattice translations, that is, space-group operations (*R*, **t**) acting on fractional coordinates. In practice, augmented pairs are generated by applying point and space-group operations identified from the relaxed structures, subsequently remapping the atoms onto the voxel grid using the same periodic boundary conditions as defined in eq 2. This yields input/target pairs consistent with the crystal symmetry and avoids the introduction of unphysically rotated cells.

### 2.7 Data Sets and Data Acquisition

We selected CH_4_ and CO_2_ as molecular benchmarks because they serve a specific dual purpose. First, they represent fundamental chemical building blocks with distinct bonding environments (tetrahedral sp^3^ and linear sp), providing a baseline for the physical consistency. Second, the use of trajectories generated via ab initio molecular dynamics (AIMD) allows us to evaluate the model’s sensitivity to continuous structural distortions. Unlike static equilibrium geometries, these thermal fluctuations provide a rigorous test for the model’s ability to capture electronic rearrangements under small structural perturbations, which is important for low-latency, interactive analysis. To ensure broader generalizability beyond these fundamental systems, the framework is further challenged with the heterogeneous crystalline environments of the Materials Project data set.

The focus on carbon-containing structures within the Materials Project subset was chosen to evaluate the framework for complex crystalline geometries. This data set includes intricate organic crystals and frameworks with large unit cells and nontrivial symmetries, providing a demanding test bed for generalization across bonding patterns and long-range spatial correlations.

The first data set consists of 852 snapshots extracted from AIMD simulations of the CO_2_ molecule, each consisting of paired atomic positions (POSCAR file) and volumetric charge density (CHGCAR file). The initial CO_2_ gas-phase configurations were sampled using AIMD within the canonical ensemble (NVT) at 300 K. The equations of motion were integrated using a Langevin thermostat with a time step of 1 fs over a production run of 20 ps. For this phase, a kinetic energy cutoff of 450 eV was used, and the electronic convergence was set at 10^–5^ eV to ensure an efficient but accurate exploration of the molecular phase space.

To generate the charge-density database, we extracted snapshots from the AIMD trajectory and subjected them to rigorous static first-principles calculations. To satisfy the high-accuracy requirements of gas-phase molecular systems, the energy cutoff increased to 850 eV, ensuring a complete basis set for carbon valence electrons. The electronic SCF loop converged to 10^–6^ eV, and we eliminated FFT grid aliasing and included nonspherical contributions to the PAW gradients. Symmetry was disabled to avoid constraints on the electronic distribution of the dynamic molecular configurations.

The second data set contains 1000 snapshots of the CH_4_ molecule, obtained analogously through AIMD simulations, likewise comprising the complete set of geometric and volumetric data for each configuration. These data sets collectively encompass both molecular and periodic systems, spanning a range of structural configurations and charge density patterns relevant to data-driven electronic structure prediction.

The third data set, consisting of 1104 carbon-containing structures, was retrieved from the Materials Project database via API, with their volumetric electronic distributions represented on real-space grids as CHGCAR files.

In this work, models are trained and evaluated *within* each chemical/structural domain (Materials Project C-containing crystals, CH_4_ AIMD, and CO_2_ AIMD). Unless explicitly stated otherwise, the reported “test” performance is therefore *in-distribution* (IID) with respect to the data set construction and split procedure for that domain. In particular, we do not claim that a model trained on one domain (e.g., CH_4_) will directly transfer to a chemically distinct domain (e.g., crystals or CO_2_) without additional training. Such out-of-distribution applications inherently introduce high predictive uncertainties, because both the density magnitude distribution and the underlying bonding motifs can change substantially across compositions, phases, and thermodynamic conditions. Rigorously quantifying this epistemic uncertainty for cross-domain transfer is beyond the scope of the current methodology, which strictly focuses on in-distribution reliability.

## 3 Results and Discussion

In this section, we evaluate the performance of the proposed Struct2ML pipeline in predicting 3D electronic charge densities from voxelized multichannel representations of atomic structures. Across all experiments, atomic configurations were embedded into fixed 60^3^ voxel grids using a 12-channel physical descriptor tensor. Model optimization employed a weighted multiobjective loss function integrating MSE, projected MSE, RMSE, relative error, structural similarity (SSIM), and Dice-based spatial overlap terms. Unless explicitly stated otherwise, MAE and RMSE are computed on the *denormalized* charge density (after inverting the [0, 1] scaling used during training) and are reported in units of *e*/Å^3^, whereas relative error (eq 14) and *R* ^2^ are dimensionless.

We report performance through quantitative metrics, including mean errors and *R* ^2^ scores. The model’s efficacy was systematically tested across three diverse data sets detailed on the previous section: (i) snapshots of the CO_2_ molecule extracted from ab initio molecular dynamics providing a comprehensive set of paired geometric and volumetric data (POSCAR and CHGCAR files); (ii) snapshots of the CH_4_ molecule obtained in an analogous manner to the previous case, and (iii) a curated set of carbon-containing crystalline structures retrieved from the Materials Project. Together, these benchmarks assess the model’s robustness and generalizability across molecular, periodic crystalline, and dynamically perturbed systems.

The performance metric distributions for the validation and test sets show a strong overlap. To evaluate the generalization performance of the model, we analyzed both validation and test sets jointly as out-of-sample (OOS) data as well as separately to assess consistency across splits. OOS refers to the union of the validation and test sets.

### 3.1 Prediction Performance on CO_2_ Database

We evaluated the proposed 3D U-Net model on a data set of 852 CO_2_ snapshots and their corresponding charge-density volumes obtained from first-principles calculations. All structures were voxelized into 12-channel atomic-property grids of size 60 × 60 × 60, and training was performed using the weighted multiobjective *Combined Loss* described previously. The training used 614 samples, with 153 validation views and 85 independent test views.

Across the full training and test distributions, the model achieved robust and generalizable performance, with a mean relative error of ∼0.20% and a median of 0.20%, indicating highly accurate global predictions. The *R* ^2^ distribution remains tightly concentrated (median = 0.996), indicating that the model captures the dominant voxel-wise variance of the reference densities; however, *R* ^2^ alone does not validate the fidelity of properties derived from ρ­(**r**) (e.g., energies) without dedicated downstream benchmarks. We summarize these results in Table 2. Although MAE and RMSE exhibit broader variability, the interquartile ranges remain well-controlled, suggesting stable performance across diverse samples. The slope values remain close to unity, confirming minimal systematic bias.

**Table 2:** Summary Statistics of Out-of-Sample (OOS) Performance^a^

metric	rel. error (%)	MAE (*e*/Å^3^)	RMSE (*e*/Å^3^)	*R* ^2^	slope
mean	0.20	64.37	577.30	0.994	0.994
Std	0.08	33.16	310.48	0.006	0.017
min	0.01	11.28	108.38	0.963	0.939
25%	0.15	39.41	350.83	0.991	0.989
median	0.20	58.74	501.33	0.996	0.996
75%	0.25	85.64	766.21	0.998	1.001
max	0.44	176.35	1679.34	0.999	1.055

a Descriptive statistics for error and performance metrics computed over the combined validation and test sets (*N* = 238). The model exhibits low relative error and high *R* ^2^ with limited dispersion, indicating consistent predictive accuracy. Slope values close to unity suggest minimal systematic bias across samples.

While the 60^3^ grid resolution introduces a discretization trade-off that may affect the absolute numerical convergence for integrated electronic properties, it effectively captures the spatial topology and relative charge distribution across diverse bonding environments. This level of granularity was benchmarked to prioritize high-throughput performance and low-latency analysis over absolute thermochemical precision. As demonstrated in the Supporting Information (Section 2 and Figure S5), this resolution represents a practical balance between physical representation and computational efficiency, providing a robust surrogate for rapid materials screening and interactive analysis.

The high overlap between predicted and calculated densities (*R* ^2^ > 0.98) indicates strong voxel-level agreement and preservation of key spatial features in the volumetric charge distribution. This supports the use of Struct2ML as a fast diagnostic signal and for downstream visualization; validation of derived quantities (e.g., energies or nonself-consistent field (NSCF) observables) requires additional task-specific benchmarks.

The distributions of the principal performance metrics for the validation and test sets are shown in Figure 4. Across all evaluated metrics, including relative error, MAE, and *R* ^2^, both subsets exhibit highly overlapping distributions with comparable central tendencies and dispersion profiles. The relative error distributions remain narrowly concentrated around low values for both partitions, while the MAE distributions display similar right-skewed behavior with only limited deviation in the upper tails. Likewise, the *R* ^2^ distributions are strongly concentrated near unity, indicating a consistently high reconstruction fidelity across unseen samples.

**Figure 4:**
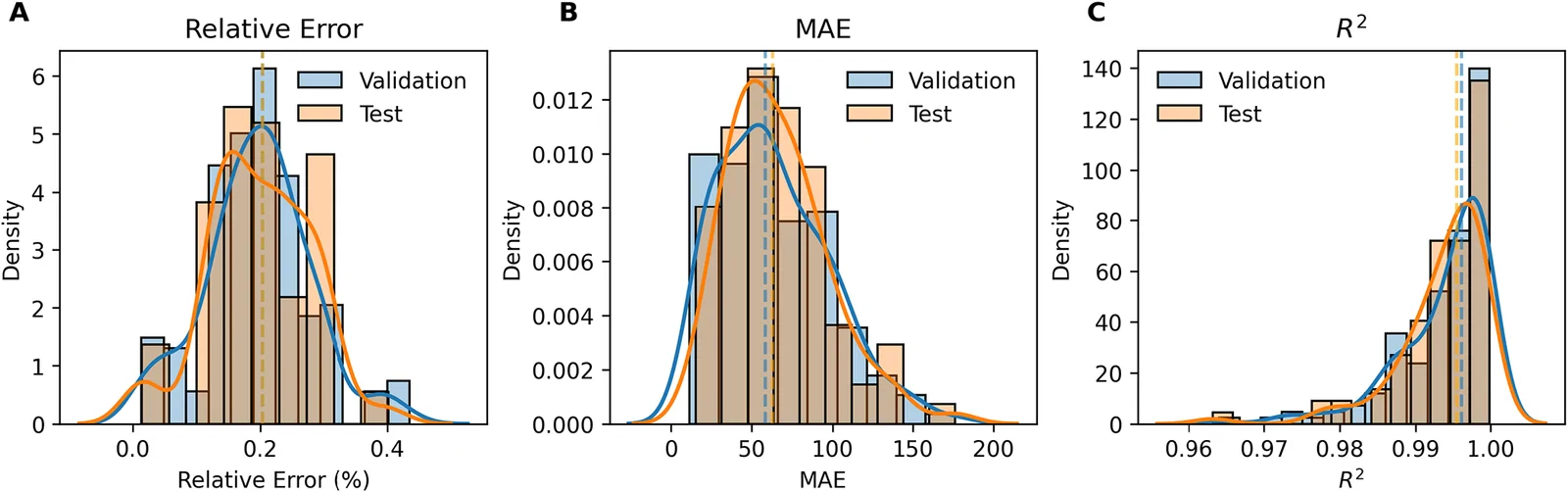
Comparison of validation and test performance distributions. Overlaid histograms and kernel density estimates for validation and test sets. (A) Relative error (%), (B) MAE, and (C) *R* ^2^. Dashed lines indicate median values for each split. The strong overlap between validation and test distributions indicates consistent generalization performance and suggests minimal overfitting or data set shift.

The close agreement between validation and test distributions suggests that the model maintains stable predictive behavior under independent data partitions with no evidence of substantial performance degradation or distributional shift between subsets. In particular, the similarity of the density curves and median values across metrics indicates robust generalization and limited overfitting, reinforcing the reliability of learned volumetric representations.

Figure 5 summarizes the aggregate out-of-sample (OOS) performance obtained by combining the validation and test sets into a unified evaluation distribution. The relative error distribution remains strongly concentrated at low values, with most samples clustered below 0.30%, indicating consistently accurate volumetric reconstructions across unseen configurations. Similarly, the MAE distribution is centered around moderate values with limited dispersion, although a small number of higher error cases contribute to a modest right-tailed behavior.

**Figure 5:**
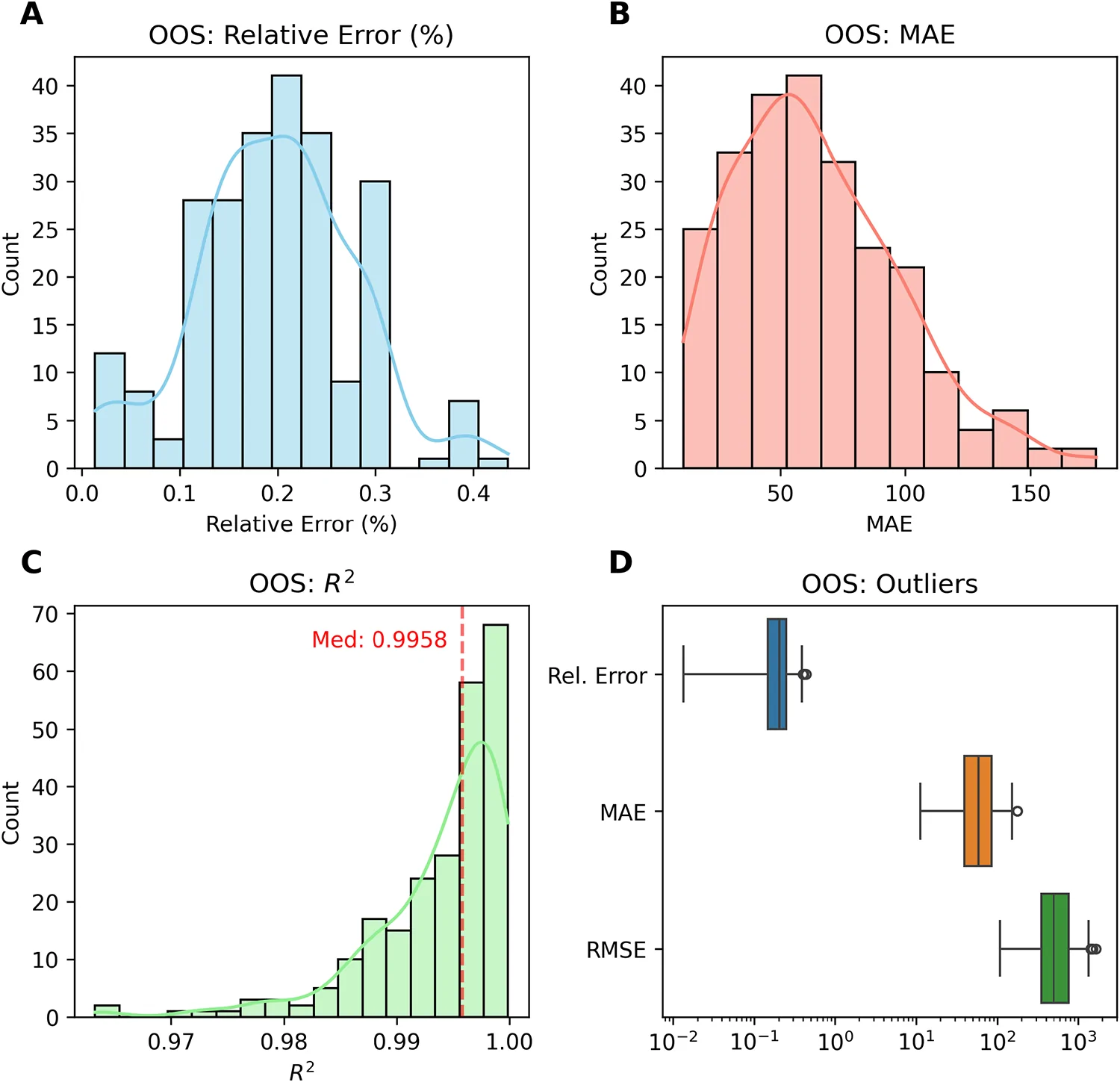
Out-of-sample (OOS) performance distribution. Distribution of model errors across the combined validation and test sets. (A) Relative error (%) distribution with kernel density estimate. (B) Mean absolute error (MAE) distribution. (C) Coefficient of determination (*R* ^2^), showing strong concentration near unity; the dashed line indicates the median value. (D) Boxplot summarizing the spread and outliers of relative error, MAE, and RMSE (log scale). Together, these results indicate high predictive accuracy with limited dispersion and a small number of outlier cases.

The *R* ^2^ distribution is sharply concentrated near unity, with a median value of approximately 0.996, indicating that the predicted charge density fields preserve the dominant voxel-wise spatial variance of the reference DFT densities across the majority of samples. Importantly, even the lower tail of the *R* ^2^ distribution remains within a high-performance regime, suggesting that substantial reconstruction failures are rare within this in-distribution of the CO_2_ setting. Boxplot analysis further confirms the overall stability of the framework. Relative error and MAE exhibit relatively compact interquartile ranges with only a limited number of outliers, whereas RMSE shows broader variability due to its increased sensitivity to localized high-intensity deviations within volumetric density fields.

#### 3.1.1 Out-of-Sample Performance Analysis (CO_2_)

We evaluated the model on the combined validation and test sets (OOS), comprising 238 samples. Overall, the model exhibits highly accurate and consistent predictive performance. The relative error remains very low, with a mean of 0.20% and median of 0.20%, indicating excellent agreement in the integrated charge density. The distribution is narrow, with most samples concentrated between ∼0.15 and 0.25%, and a maximum error below 0.44%, reflecting strong global conservation across all predictions.

In contrast, absolute error metrics (MAE and RMSE) display broader variability, with mean values of 64.4 and 577.3, respectively. This suggests that while local deviations may vary in magnitude across samples, they do not significantly affect global accuracy. The wider spread in RMSE, in particular, indicates sensitivity to localized discrepancies in high-density regions. Despite this variability, the coefficient of determination remains consistently high, with a mean *R* ^2^ of 0.994 and median of 0.996. Most samples cluster near unity, indicating that the model captures the dominant spatial variance of the charge density at the voxel level; however, *R* ^2^ should not be interpreted as a direct validation of downstream physical observables derived from ρ­(**r**). Even the lowest observed value (0.963) still indicates strong predictive performance on this data set.

#### 3.1.2 Extreme Cases Analysis (CO_2_)

Analysis of extreme cases reveals important insights into the model’s behavior. When ranked by relative error, the best-performing sample (snapshot 4990) achieves an error of only 0.01%, indicating near-perfect conservation of integrated quantities. The worst case (snapshot-3370), while higher (0.44%), still maintains a strong *R* ^2^ (0.994) and comparable MAE/RMSE values. This suggests that differences in the relative error are driven primarily by small global scaling effects rather than structural inaccuracies.

When considering *R* ^2^, the best-performing case (snapshot 2220) achieves near-perfect agreement (*R* ^2^ = 0.999) with very low MAE and RMSE, reflecting both spatial and quantitative accuracy. In contrast, the worst *R* ^2^ case (snapshot 5970) shows a noticeable drop (0.963) and significantly larger MAE (176.2) and RMSE (1475.9). Notably, its relative error remains low (0.13%), indicating that the global magnitude is preserved while local spatial deviations dominate the error.

These results highlight a clear separation between the global and local performance characteristics. The consistently low relative error demonstrates that the model reliably preserves integrated physical quantities, while the high *R* ^2^ values confirm strong agreement in the spatial structure. Differences between the two metrics indicate that localized discrepancies can occur without substantially affecting the global accuracy.

Overall, the model exhibits robust generalization across unseen samples, achieving both high structural fidelity and strong conservation properties even in the presence of localized variations.

### 3.2 CH_4_ Database

Across the OOS data set, the model achieves a mean relative error of ∼1.02% and a median of 0.75% (Table 3), indicating low overall deviation between predicted and true charge densities. The *R* ^2^ distribution is tightly concentrated (median = 0.994), reflecting strong agreement at the spatial level. The narrow interquartile range across all metrics suggests stable performance, while the slope values remaining close to unity confirm the absence of systematic over- or underestimation. Although a small number of higher-error cases are observed (max relative error ≈ 4.6%), these represent a limited fraction of the data set.

**Table 3:** Summary Statistics of Out-of-Sample (OOS) Performance for CH_4_ ^a^

metric	rel. error (%)	MAE (*e*/Å^3^)	RMSE (*e*/Å^3^)	*R* ^2^	slope
mean	1.02	37.72	294.20	0.993	0.999
Std	0.80	10.96	84.20	0.004	0.015
min	0.01	15.40	111.77	0.973	0.944
25%	0.43	29.87	228.97	0.991	0.991
median	0.75	37.15	293.40	0.994	1.000
75%	1.46	44.02	343.40	0.996	1.010
max	4.61	77.19	587.86	0.999	1.036

a Descriptive statistics for error and performance metrics computed over the combined validation and test sets (*N* = 280). The model achieves low relative error and high *R* ^2^ across the dataset, with narrow dispersion, indicating consistent predictive accuracy. The slope remains close to unity, suggesting minimal systematic bias.

The distributions of the principal performance metrics for the validation and test sets are listed in Figure 6. Across relative error, MAE, and *R* ^2^, both subsets exhibit highly overlapping distributions with closely aligned density profiles and central tendencies, indicating strong consistency between validation and test performance. The relative error distributions are concentrated at low values, with the majority of samples remaining below approximately 2%, although a moderate right-skewed tail is observed due to a limited number of higher-error configurations. Similarly, the MAE distributions display nearly identical shapes across both subsets, centered around intermediate values with only minor deviations in the upper tails.

**Figure 6:**
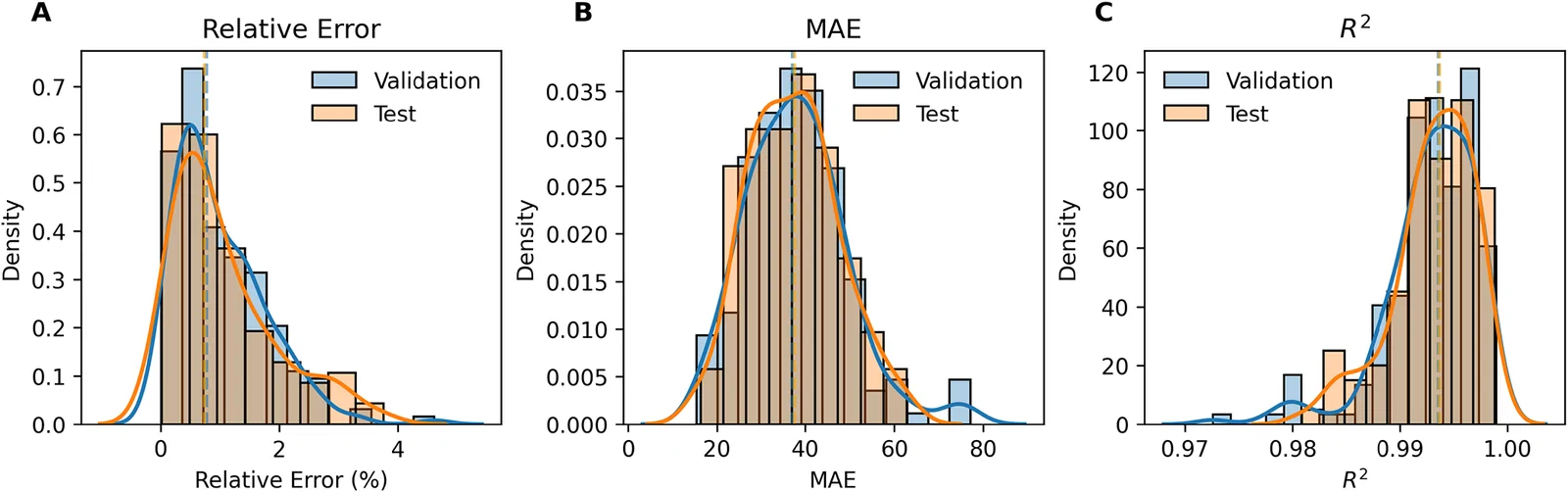
Comparison of validation and test performance distributions for CH_4_. Overlaid histograms and kernel density estimates for validation and test sets. (A) Relative error (%), (B) MAE, and (C) *R* ^2^. Dashed vertical lines indicate the median values for each split. The strong overlap between validation and test distributions across all metrics indicates consistent model behavior and minimal distributional shift.

The *R* ^2^ distributions remain strongly concentrated near unity, demonstrating that the predicted charge-density fields preserve the dominant spatial variance of the reference DFT densities across unseen structures. Importantly, the strong overlap between validation and test density curves, as well as the similarity of the median values across metrics, suggests that the model maintains stable predictive behavior without substantial evidence of overfitting or distributional shift between partitions. These results support the robustness and generalizability of the learned volumetric representations across the independent data subsets.

Figure 7 summarizes the aggregate out-of-sample (OOS) performance obtained by combining the validation and test sets into a unified evaluation distribution. The relative error distribution remains concentrated at low values, with most samples clustered below approximately 2%, although a pronounced right-skewed tail extends toward higher-error cases. This behavior indicates that the framework achieves consistently accurate reconstructions for the majority of unseen configurations while retaining a limited number of more challenging samples associated with an elevated prediction error.

**Figure 7:**
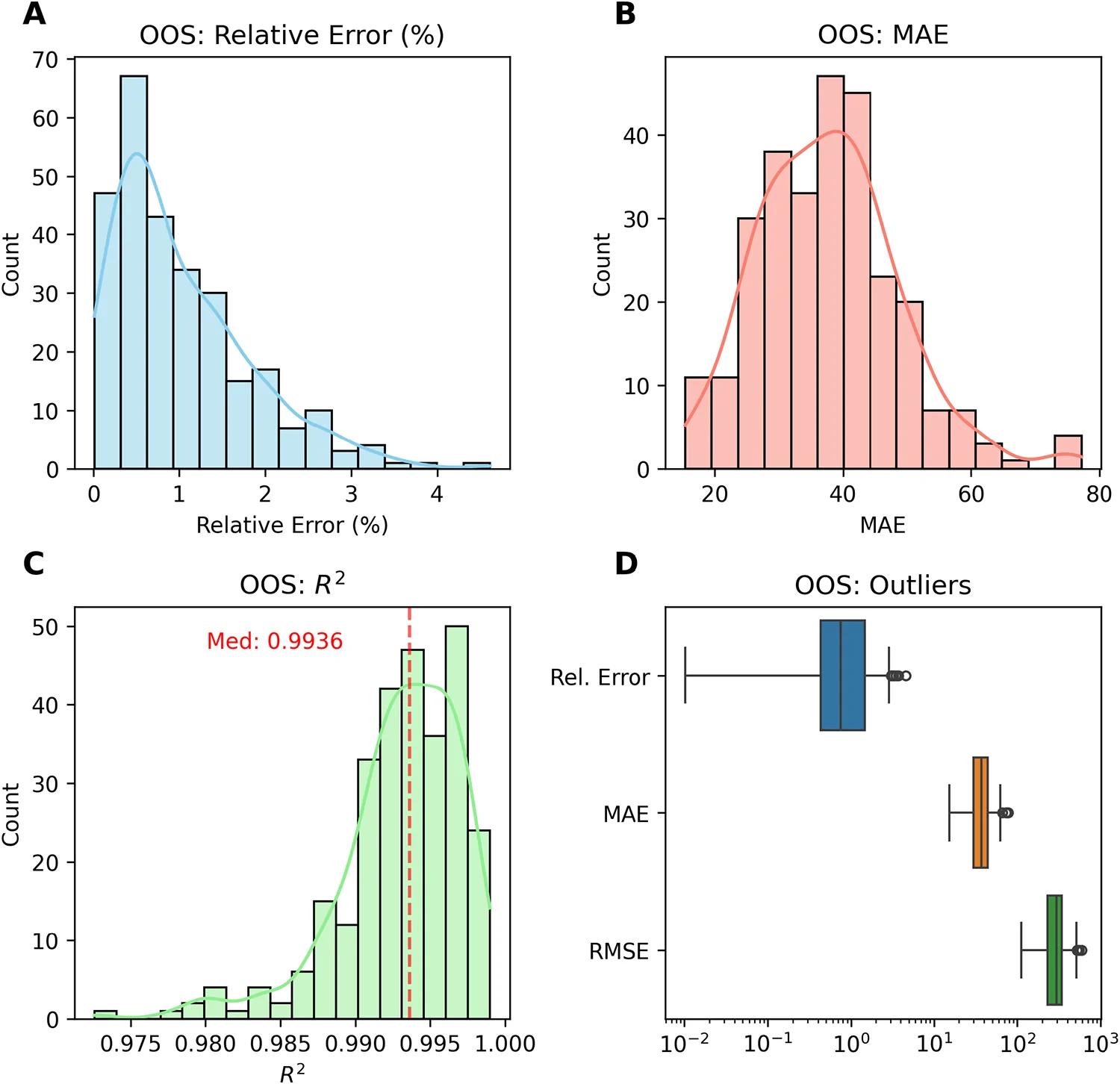
Out-of-sample (OOS) performance distribution for CH_4_. Distribution of model errors across the combined validation and test sets (OOS). (A) Relative error (%) with kernel density estimate, showing a right-skewed distribution. (B) Mean absolute error (MAE), exhibiting moderate dispersion across samples. (C) Coefficient of determination (*R* ^2^), with values tightly clustered near unity; the dashed line indicates the median (*R* ^2^ = 0.9936). (D) Boxplots summarizing the spread and outliers of relative error, MAE, and RMSE (log scale).

The MAE distribution exhibits moderate dispersion with a well-defined central peak, suggesting stable reconstruction quality across the data set. Likewise, the *R* ^2^ distribution remains sharply concentrated near unity, with a median value of approximately 0.994, indicating that the predicted volumetric charge density fields preserve the dominant spatial characteristics of the reference DFT densities across most out-of-sample structures. Even the lower tail of the *R* ^2^ distribution remains within a high-performance regime, demonstrating robust predictive alignment despite structural variability. The boxplot analysis further highlights the stability of the framework across the metrics. Relative error and MAE exhibit relatively compact interquartile ranges with only a limited number of outliers, whereas RMSE displays broader variability due to its sensitivity to localized high-intensity deviations within volumetric density fields.

#### 3.2.1 Out-of-Sample Performance Analysis (CH_4_)

To assess generalization, we evaluated the model on the combined validation and test sets, hereafter referred to as out-of-sample (OOS) data. Across 280 samples, the model demonstrates consistently high predictive accuracy. The relative error remains low overall, with a mean of 1.02% and median of 0.75%, indicating that most predictions closely match the ground truth. The distribution is moderately right-skewed, with the majority of samples below ∼1.5% error and a small number of higher-error outliers (maximum 4.61%).

Absolute error metrics further support this behavior. The mean absolute error (MAE) is 37.7, while the root mean squared error (RMSE) averages 294.2, reflecting stable performance across spatial charge distributions. The relatively narrow interquartile ranges (MAE: 29.9–44.0; RMSE: 229.0–343.4) indicate limited dispersion and good consistency across samples. Model fidelity is particularly evident in the coefficient of determination, with a mean *R* ^2^ of 0.993 and a median of 0.994. Most samples cluster near unity, confirming strong agreement between predicted and true charge densities. Even the worst-performing case in terms of *R* ^2^ maintains a high value (0.973), suggesting that the model rarely fails catastrophically.

#### 3.2.2 Extreme Cases Analysis (CH_4_)

To better understand the model behavior, we examined extreme cases based on both relative error and *R* ^2^.

The best case in terms of relative error (snapshot-2065) achieves an exceptionally low error of 0.01% while still maintaining a high *R* ^2^ (0.996), indicating near-perfect reconstruction of the spatial distribution. Conversely, the worst relative error case (snapshot-5395) reaches 4.61% yet still retains a strong *R* ^2^ (0.991). This suggests that even when the global error increases, the model preserves the overall structural relationship between predicted and true values.

When ranking by *R* ^2^, the best-performing sample (snapshot-2555) achieves *R* ^2^ = 0.999, with a low MAE and RMSE, reflecting an almost exact match. The worst *R* ^2^ case (snapshot 6660), although lower (0.973), still demonstrates substantial predictive power, with moderate increases in MAE (77.2) and RMSE (587.9).

Together, these results indicate that the model generalizes robustly across unseen data, maintaining high accuracy and stability. Importantly, discrepancies between relative error and *R* ^2^ extremes highlight complementary aspects of performance: while relative error captures global deviations, *R* ^2^ reflects the preservation of the spatial structure. The consistently high values across both metrics suggest that the model not only predicts accurate magnitudes but also faithfully reproduces the underlying patterns in the data.

### 3.3 Materials Project Database

To assess the generalization capability of the model, we evaluated its performance on out-of-sample (OOS) data by combining the validation and test sets. The results are summarized in Table 4 and visualized in Figures 8 and 9, which together provide a comprehensive view of both central tendencies and distributional behaviors across key metrics. While the validation and test distributions remain consistent, indicating stable generalization, the OOS analysis reveals increased variability and the presence of challenging cases, highlighting important limitations in the model robustness across more complex samples.

**Figure 8:**
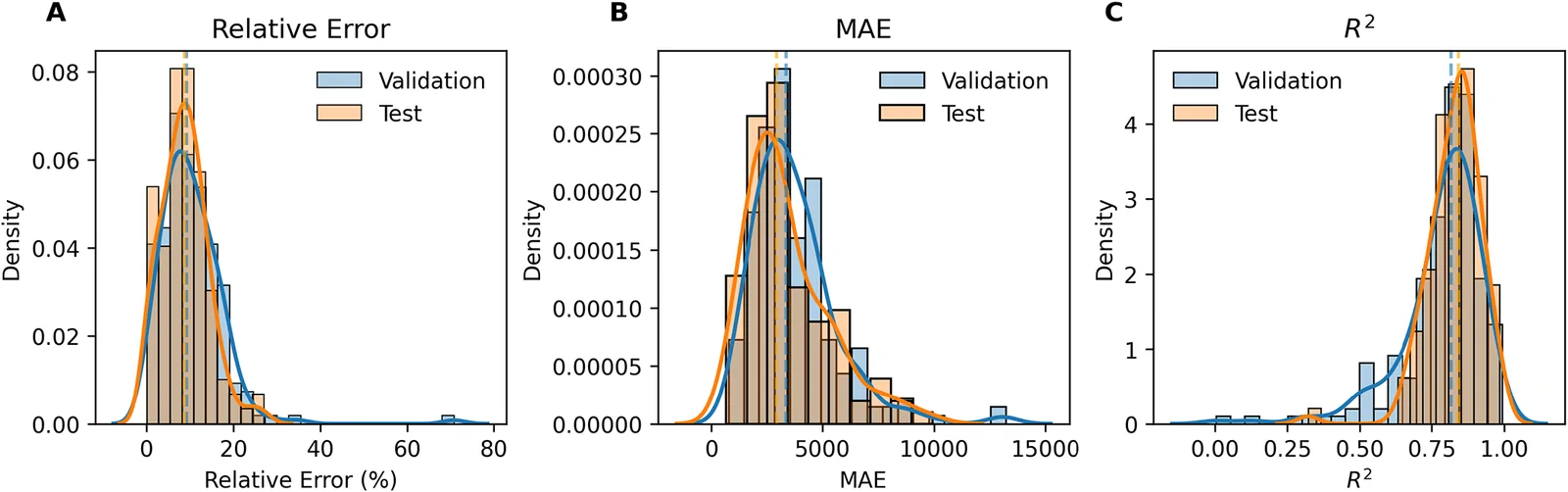
Comparison of validation and test performance distributions for the Materials Project. Overlaid histograms and kernel density estimates for (A) relative error (%), (B) MAE, and (C) *R* ^2^. Dashed vertical lines indicate the median values. The overlap suggests consistent behavior across splits despite the overall increase in error magnitude.

**Figure 9:**
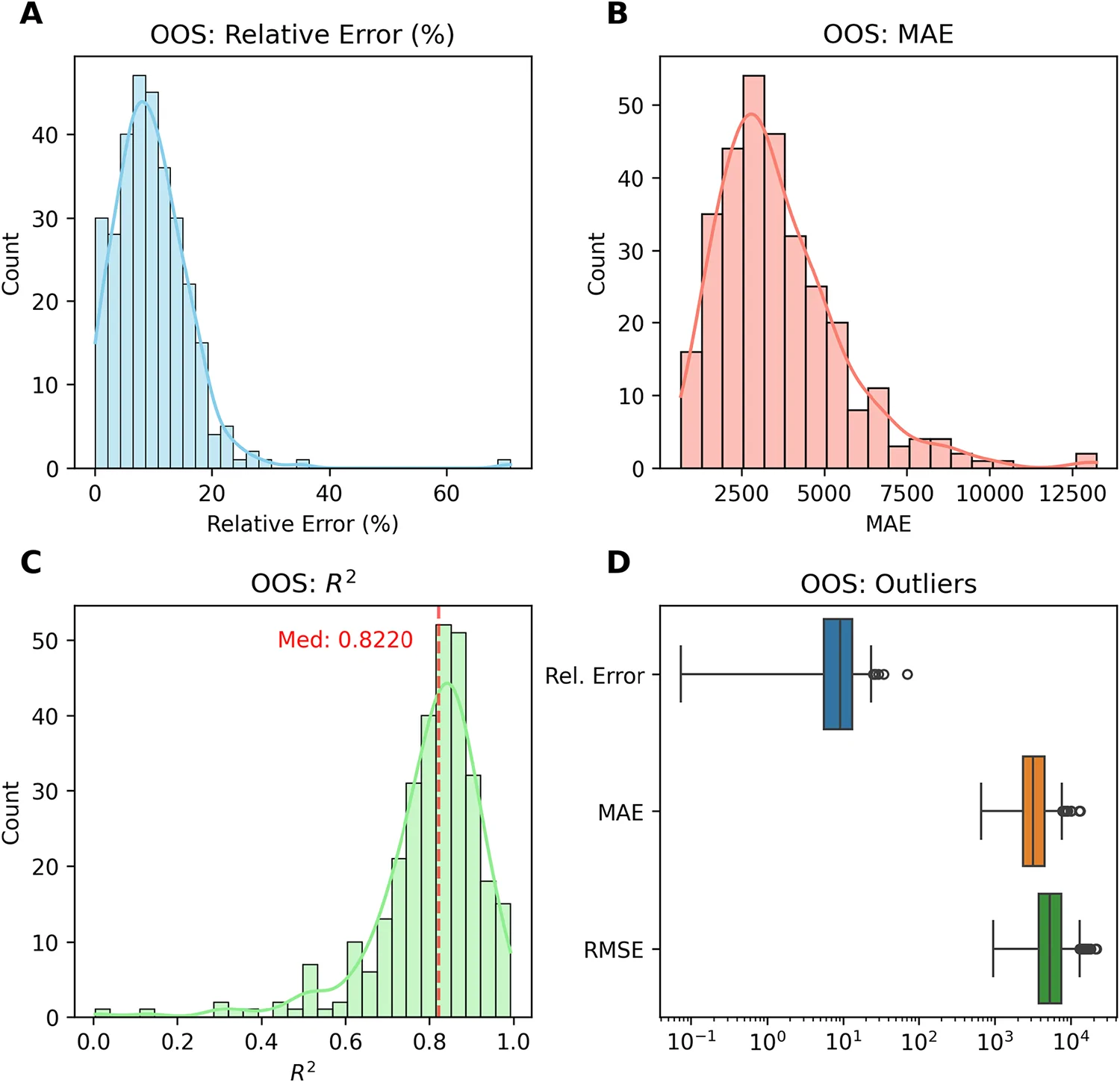
Out-of-sample (OOS) performance for the Materials Project data set. (A) Distribution of relative error (%) showing a right-skewed profile with a long tail. (B) Distribution of mean absolute error (MAE). (C) Distribution of the coefficient of determination (*R* ^2^), with a median of 0.822 (red dashed line). (D) Boxplots of Relative Error, MAE, and RMSE (log scale), emphasizing the presence of extreme outliers and wide dispersion.

**Table 4:** Summary Statistics of Out-of-Sample (OOS) Performance for the Materials Project Dataset^a^

metric	rel. error (%)	MAE (*e*/Å^3^)	RMSE (*e*/Å^3^)	*R* ^2^	slope
mean	9.78	3619.99	6097.43	0.799	0.838
Std	6.74	1918.02	3419.05	0.131	0.148
min	0.07	660.95	955.68	0.005	–0.120
25%	5.63	2356.83	3769.07	0.756	0.764
median	9.12	3200.41	5282.74	0.822	0.855
75%	13.10	4526.24	7502.83	0.879	0.921
max	70.89	13226.99	21860.72	0.993	1.369

a Descriptive statistics for error and performance metrics computed over the combined validation and test sets (*N* = 308). The model exhibits higher variability and reduced overall accuracy compared to simpler systems, with significant dispersion in *R* ^2^ and slope values.

The distributions of the principal performance metrics for the validation and test sets are listed in Figure 8. Across relative error, mean absolute error (MAE), and *R* ^2^, both subsets exhibit highly overlapping distributions with closely aligned density profiles and central tendencies, indicating strong consistency between validation and test performance.

The relative error distributions (Figure 8A) are highly concentrated at low values, with the majority of samples remaining below approximately 20%. A pronounced right-skewed tail extends toward higher values, driven by a limited number of challenging structural configurations. Similarly, the MAE distributions (Figure 8B) display nearly identical shapes across both subsets, centered around approximately 3000–4000 with minor matching deviations in the upper tails.

The *R* ^2^ distributions (Figure 8C) remain strongly concentrated near unity, with the peak density situated around 0.822. This demonstrates that the predicted charge density fields accurately preserve the dominant spatial variance of the reference DFT densities across unseen structures.

Importantly, the exceptional overlap between the validation and test density curves, alongside the tight alignment of their respective mean/median indicators (represented by the dashed vertical lines), suggests that the model maintains a stable predictive behavior. The lack of divergence between these two independent partitions confirms that the model does not suffer from overfitting or a distributional shift. Consequently, these results support the robustness and generalizability of the learned volumetric representations across the independent data subsets.

Figure 9 summarizes the aggregate out-of-sample (OOS) performance for the Materials Project data set obtained by combining validation and test samples into a unified evaluation distribution. In contrast to more chemically constrained molecular systems, the relative error distribution exhibits substantially broader dispersion and pronounced right-skewed behavior, with a subset of samples extending toward significantly larger reconstruction errors. This behavior reflects the intrinsic structural and compositional heterogeneity of the Materials Project database, which encompasses a wide diversity of crystal symmetries, bonding environments, atomic densities, and electronic configurations.

The MAE distribution similarly displays elevated variance and an extended upper tail, indicating that the reconstruction difficulty increases considerably for certain complex crystalline systems. Nevertheless, the majority of samples remain concentrated within moderate error ranges, suggesting that the framework preserves stable predictive behavior across a large portion of the data set despite the increased complexity. Importantly, the *R* ^2^ distribution remains strongly biased toward high values, with a median of approximately 0.822, indicating substantial voxel-level agreement with the reference DFT volumetric densities for many out-of-sample structures; however, this statistic does not, by itself, certify the accuracy of downstream-derived observables without explicit validation.

The boxplot analysis further illustrates the broader variability observed in the Materials Project benchmark relative to the molecular data sets. Relative error, MAE, and RMSE all exhibit wider interquartile ranges and a larger number of outliers, reflecting the presence of particularly challenging materials with highly diverse electronic environments. RMSE demonstrates the largest spread due to its sensitivity to localized high-density deviations and accumulated volumetric reconstruction errors. Collectively, these results indicate that although predictive variability increases substantially in large-scale heterogeneous material data sets, Struct2ML maintains robust volumetric reconstruction capability and meaningful predictive alignment across a broad range of crystalline systems.

#### 3.3.1 Out-of-Sample Performance Analysis (Materials Project)

We evaluated the model on the combined validation and test sets (OOS) comprising 308 samples. In contrast to the previous systems, performance on this data set shows substantially higher variability and reduced overall accuracy. The relative error exhibits a broad distribution, with a mean of 9.78% and a median of 9.12%, indicating significant deviations between predicted and true integrated charge densities. While a subset of samples achieves low error (<1%), the distribution is strongly right-skewed, with extreme values reaching up to 70.9%, revealing the presence of severe failure cases.

Absolute error metrics are correspondingly large, with mean MAE and RMSE values of 3619.99 and 6097.43, respectively. The wide interquartile ranges (MAE: 2356.8–4526.2; RMSE: 3769.1–7502.8) and high standard deviations indicate substantial heterogeneity across samples, suggesting that model performance is highly system dependent. This variability is also reflected in the coefficient of determination. The mean *R* ^2^ drops to 0.799, with a large standard deviation (0.131) and a minimum value close to zero (0.005), indicating near-complete loss of predictive power in some cases. Although the upper range remains high (up to 0.993), the overall distribution suggests inconsistent reconstruction of spatial charge distributions.

#### 3.3.2 Extreme Cases Analysis (Materials Project)

The analysis of extreme cases highlights the model’s instability on this data set. The best case in terms of relative error (mp-572874) achieves 0.07%, demonstrating that the model is capable of highly accurate predictions under favorable conditions. However, even in this case, the *R* ^2^ (0.896) remains noticeably lower than in previous data sets, suggesting that good global agreement does not necessarily imply accurate spatial reconstruction.

The worst-performing sample (mp-1198801) represents a clear failure mode with an extremely high relative error (70.9%) and near-zero *R* ^2^ (0.005). This indicates both a breakdown in the global magnitude prediction and a complete loss of structural fidelity. Notably, this same sample is also the worst-case when ranked by *R* ^2^, confirming it as a critical outlier. When considering *R* ^2^, the best-performing sample (mp-1204151) achieves 0.993, with relatively low MAE and RMSE values, indicating accurate spatial reconstruction. However, even in this case, the relative error (2.25%) is higher than typical values observed in other data sets.

These results indicate that the model does not generalize uniformly across all of the systems. While it retains the capacity for high accuracy in certain cases, its performance is highly variable and degrades significantly for a subset of samples. The divergence between the relative error and *R* ^2^ further highlights the complexity of the task: some samples exhibit reasonable global agreement but poor spatial reconstruction, while one outlier fails in both aspects. The presence of extreme outliers suggests that the model encounters regimes that are not well represented in the training distribution.

To further investigate the internal representations learned by the network and assess whether the model captures physically meaningful spatial patterns beyond purely statistical correlations, we performed complementary explainability analyses based on gradient saliency and SHAP attribution in the latent space (Supporting Information).

### 3.4 Physical Consistency and the Accuracy-Throughput Trade-Off

To summarize the predictive performance across all evaluated regimes, the preservation of key global constraints is evidenced by metrics from different chemical environments. In molecular data sets, the model demonstrates near-perfect charge conservation, with median relative errors of 0.20% for CO_2_ (*R* ^2^ = 0.996) and 0.75% for CH_4_ (*R* ^2^ = 0.994). For the high-dimensional and heterogeneous space of the Materials Project, the performance is lower and more variable, with a median *R* ^2^ of 0.822.

Slope values range from 0.855 to 1.000, indicating the approximate preservation of integrated charge and limited systematic bias, with substantially better consistency in the molecular data sets than in the heterogeneous Materials Project subset. These results indicate that the multiobjective loss function, particularly the projection-based MSE, helps regularize the network toward density fields with improved integrated-charge consistency.

These metrics should be interpreted in the context of the accuracy–throughput trade-off. As shown in the comparative analysis in Section 3 of the Supporting Information, Struct2ML operates in a different accuracy regime than *E*(3)-equivariant graph neural networks (GNNs), which typically prioritize symmetry and accuracy. In exchange, Struct2ML achieves millisecond-scale inference latency (1–10 ms) even in the complex Materials Project space, enabling tight interactive and high-throughput workflows where immediate feedback may be preferable in specific exploratory or interactive settings, provided that limitations in quantitative accuracy are recognized.

Beyond visualization, the predicted charge density may potentially be explored for accelerating the first-principles workflows. Specifically, the density maps generated by Struct2ML may potentially serve as initial guesses for the electron density (*n*_in_) in density functional theory (DFT) packages with the potential to reduce the number of self-consistent field (SCF) iterations required for convergence. Furthermore, in applications where the total energy is not the primary target, the predicted density could, after appropriate validation and format conversion, be explored in nonself-consistent field (NSCF) workflows to obtain rapid estimates of electronic properties, such as the density of states (DOS). A quantitative validation of SCF acceleration, total energy accuracy, and NSCF-derived observables is outside the scope of the present work and is left for future study.

## 4 Conclusions

The present results demonstrate the capability of the proposed framework to predict full 3D electronic charge density grids directly from the structural information alone. Across distinct data sets spanning molecular dynamics trajectories and crystalline materials, the model approximately preserved integrated charge, achieved low voxelwise errors, and reproduced spatial charge-density patterns with high fidelity for the molecular data sets and with data set-dependent accuracy for the Materials Project subset.

For the CO_2_ data set, the model exhibited highly stable and accurate out-of-sample performance, achieving a mean relative error of approximately 0.20% and median *R* ^2^ values above 0.995. The narrow metric distributions and strong overlap between validation and test sets indicate robust generalization across diverse molecular configurations generated during molecular dynamics simulations. Similarly, the CH_4_ data set demonstrated strong predictive consistency, with a mean relative error of approximately 1.02% and median *R* ^2^ ≈ 0.994. Although a small number of higher-error cases were observed, the model maintained strong spatial agreement even in challenging samples, indicating the robust reconstruction of both global and local charge-density features.

In contrast, the Materials Project data set revealed substantially greater variability and reduced predictive stability. While the model remained capable of highly accurate predictions for a subset of crystalline systems, overall performance was notably lower, with a mean relative error of 9.78% and a median *R* ^2^ of 0.822. The presence of outliers and broad metric distributions suggests that the structural heterogeneity of the Materials Project systems introduces regimes that are insufficiently represented in the training distribution. These results indicate that model generalization is strongly system-dependent and that the reconstruction of charge densities in heterogeneous crystalline environments remains a considerably more challenging task than reconstruction within molecular dynamics trajectories of simpler systems.

Despite these limitations, the framework demonstrates substantially lower latency than performing a new self-consistent DFT calculation for each queried structure, although the two procedures provide different levels of physical information. Struct2ML reconstructs full 3D electronic charge densities within the millisecond range (∼1 to 10 ms per inference on GPU) in the fixed-grid setting studied here.

An extensive ablation study combined with interpretability analyses (gradient saliency and SHAP attribution) in Section 2 of the Supporting Information supports the relevance of the adopted 12-channel descriptor configuration within the tested systems. The data indicate that richer multichannel inputs improve predictive stability relative to reduced-channel baselines.

Overall, these findings highlight the effectiveness of combining a 3D U-Net architecture with a physics-informed weighted loss function for learning complex electronic density distributions from an atomic structure alone. The millisecond-scale latency, paired with strong morphological agreement in the molecular data sets, supports the potential of Struct2ML as a computationally efficient surrogate optimized for real-time electronic-structure inference and interactive visualization.

Looking toward future research directions, several avenues appear particularly promising to broaden the physical fidelity and operational integration of Struct2ML. Expanding the framework to more complex and heterogeneous systems, such as surfaces, interfaces, defective structures, and broader chemical compositions, will be important to assessing its applicability beyond the systems considered here. In particular, the system-dependent generalization observed for the heterogeneous Materials Project data set motivates systematic benchmarks across distinct chemical families to establish the transferability limits of the present representation. Coupling the predicted density fields to derived physical observables, including electrostatic potentials and topological descriptors, and systematically validating these quantities against first-principles references will provide an additional assessment of the physical fidelity of the predicted densities.

Furthermore, the millisecond-scale inference latency opens opportunities to integrate on-the-fly density predictions into molecular dynamics and active learning workflows. In this context, an appropriate uncertainty quantification strategy could be used to identify poorly represented configurations and trigger explicit DFT calculations when required. Hybrid architectures combining the computational efficiency and direct volumetric output of grid-based convolutional models with the symmetry properties and accuracy of equivariant approaches also represent a promising direction for improving the predictive performance while retaining low-latency inference. Finally, the rapid generation of three-dimensional density fields may support interactive visualization environments, including immersive VR platforms, in which structural modifications and associated predicted density rearrangements can be explored in real time.

## Supplementary Material



## Notes

Additional technical details on the Struct2ML framework. It presents an explanatory analysis comparing gradient-based and SHAP attributions, a feature ablation study validating the 12-channel descriptor choice, an expanded literature review of alternative architectures, and complete model training and convergence curves (PDF)
