# Supplementary material for: Struct2ML: End-to-End Prediction of Electronic Charge Densities Using 3D U‑Net
Source: ACS Omega. 2026 Aug 21;11(34):52123–40. doi: 10.1021/acsomega.6c07986 (PMC13625562; doi:10.1021/acsomega.6c07986)
Supplement: Supplementary file 1 [file ao6c07986_si_001.pdf]

Supplementary Material

Struct2ML: End-to-End Prediction of Electronic Charge Densities Using 3D U-Net

João Victor Vilela Cassiano, Marcel Valério de Arruda, Alvaro David Torrez-Baptista, and Caetano Rodrigues Miranda*

Universidade de São Paulo, Instituto de Física, Rua do Matão, 1371, 05508-090, São Paulo, SP

E-mail: crmiranda@usp.br

1 Explainability Analysis: Gradient vs. SHAP Attribution

To probe the internal representations learned by the 3D U-Net, we combined gradient-based saliency with SHAP-based attribution in the latent space. Gradient saliency maps were computed as the derivative of a region-integrated output with respect to the input charge density, capturing the local sensitivity of the model to perturbations. In parallel, we extracted latent feature vectors from the bottleneck layer via global spatial pooling and trained a surrogate model to approximate the mapping between latent representations and the same region-integrated target. SHAP values were computed on this surrogate model to quantify the contribution of each latent feature channel, which were subsequently projected back to the input space using activation maps. All analyses were performed on the held-out test set of the Materials Project database ($n = 110$).

1.1 Explainability Findings

Across the test set, gradient-based and SHAP-based attribution maps exhibited systematically distinct spatial characteristics (Figure S1). Gradient saliency consistently highlighted localized regions of high sensitivity, typically concentrated around sharp peaks in the predicted charge density. In contrast, SHAP maps revealed smoother and more spatially distributed patterns, indicating that the model's predictions depend on broader contextual features encoded in the latent representation.

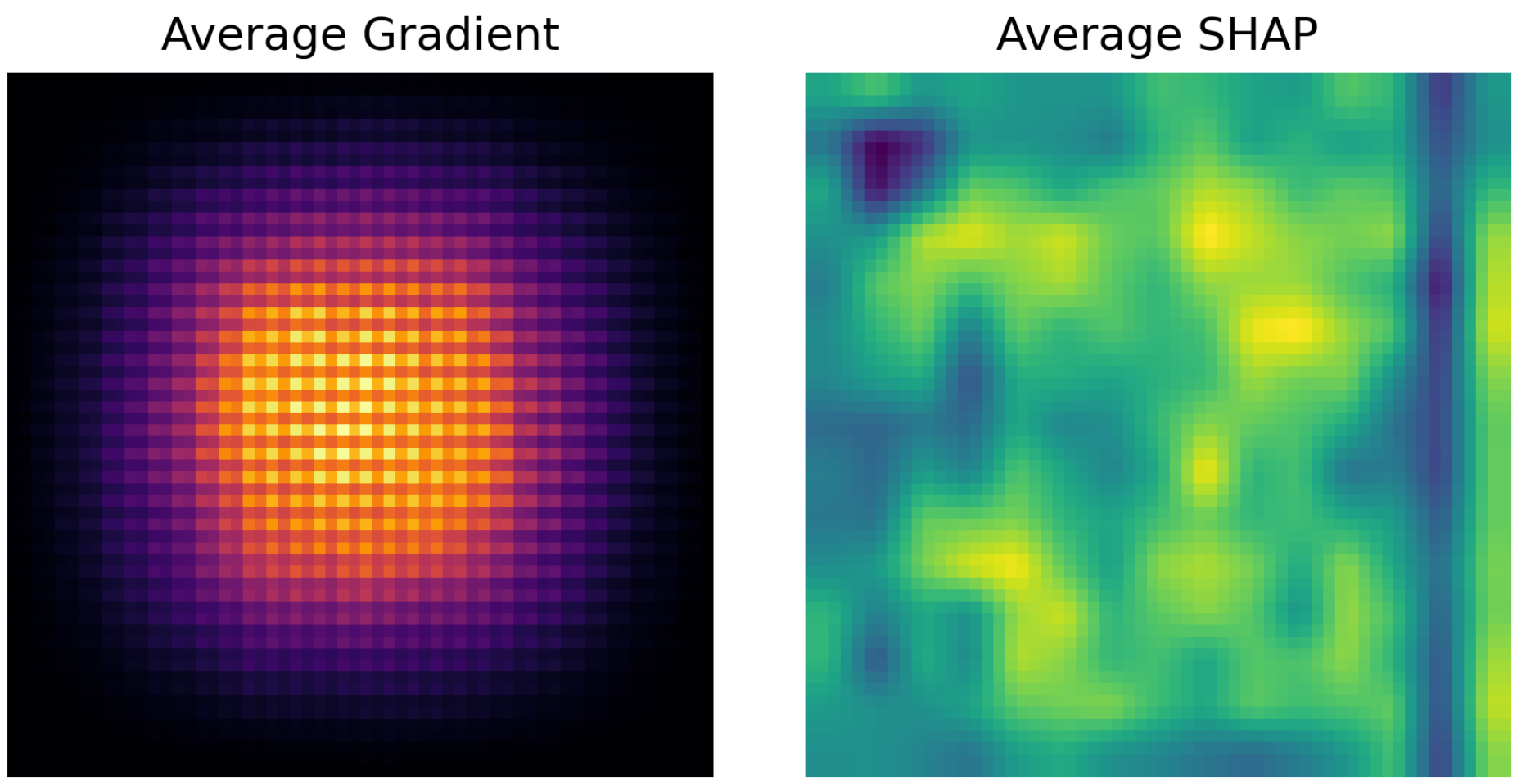

Figure S1: Average Attribution Maps. Comparison of the mean gradient saliency (left) and mean SHAP attribution (right) across the test set. The gradient map shows high local sensitivity concentrated in the center, while the SHAP map illustrates a more distributed global feature importance.

Quantitatively, the agreement between the two methods was weak on average, with a mean Pearson correlation of 0.031 ($\sigma = 0.244$) across 110 test samples (Table S1). The distribution of correlations spanned a wide range (from -0.483 to 0.572). This broad variability indicates that the relationship between gradient and SHAP explanations is highly input-dependent, suggesting that they capture complementary aspects of model behavior (Figure S2).

Table S1: Explainability Agreement Statistics. Quantitative comparison between gradient-based saliency and SHAP attribution maps across the Materials Project test set ($N = 110$). The metrics describe the distribution of Pearson correlation coefficients (r) calculated for each sample.

Metric	Value
Samples	110
Mean correlation	0.031
Std correlation	0.244
Min correlation	-0.483
Max correlation	0.572
Best r (High Correlation Case)	0.572
Worst r (Decoupling Case)	-0.004

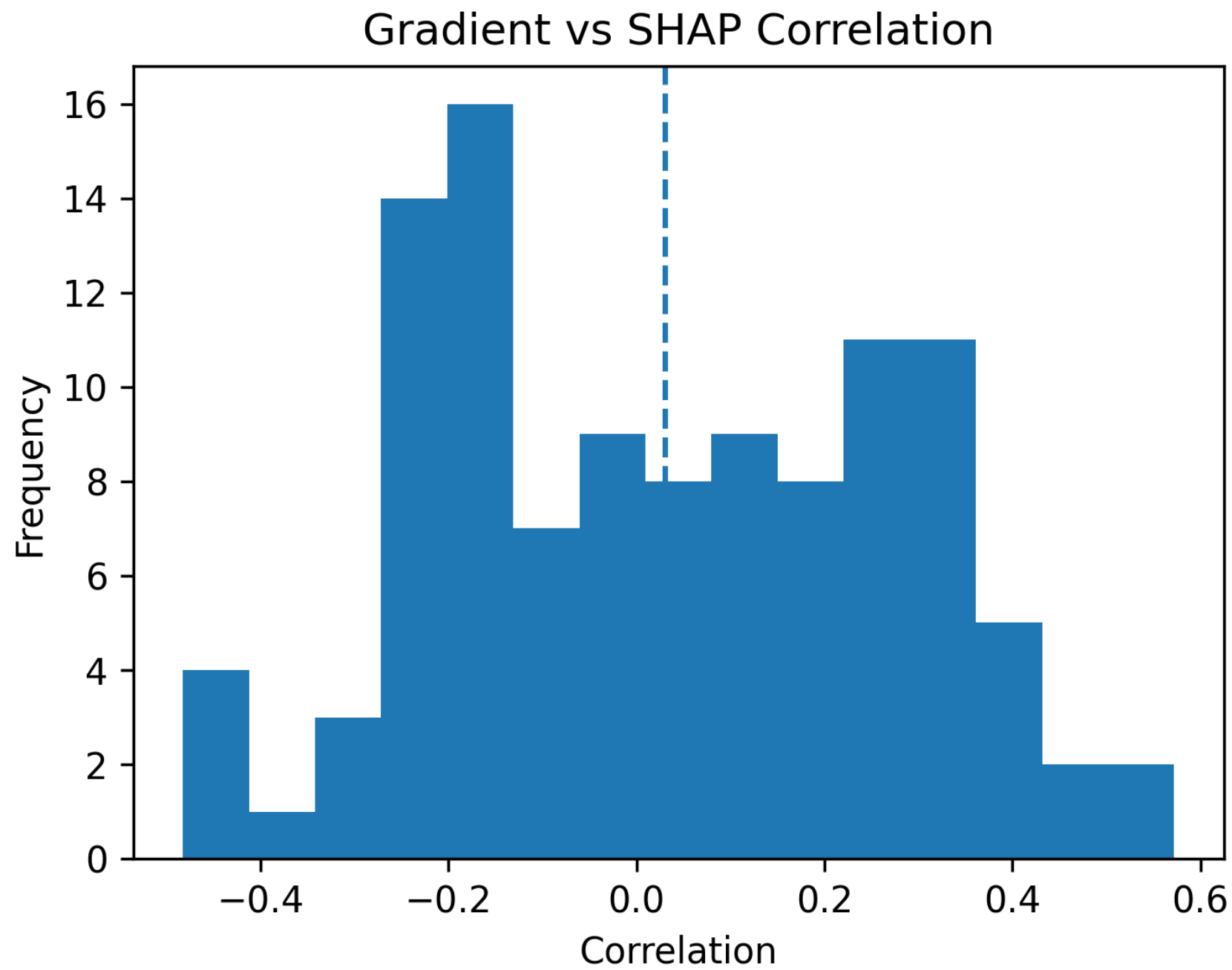

Figure S2: Distribution of Interpretability Correlations. Histogram showing the Pearson correlation coefficients (r) between gradient and SHAP attribution maps for 110 test samples. The mean correlation is near zero (0.03), with a wide variance indicating highly input-dependent alignment.

1.2 Physical Interpretation

From a physical perspective, the variability in agreement between gradient and SHAP attributions reflects the intrinsically non-local nature of electronic charge densities. While high-density regions are associated with atomic cores, the global distribution is governed by the collective arrangement of atoms, chemical identities, and the lattice environment.

1.2.1 Local Sensitivity vs. Global Contribution

The localized emphasis of gradient maps near density peaks is consistent with the model being sensitive to perturbations at atomic sites, in line with the steep spatial gradients near nuclei. Conversely, the smoother SHAP maps suggest that the model encodes distributed structural features—such as coordination environments and long-range interactions—consistent with electronic-structure theory where charge density arises from delocalized wavefunctions.

The range of observed correlations ($r \in [-0.48, 0.57]$) reveals two distinct regimes:

- **Aligned Regime ($r \approx 0.57$):** Local sensitivity and global predictive relevance coincide, highlighting regions where atomic positions dominate the output(Figure S3).

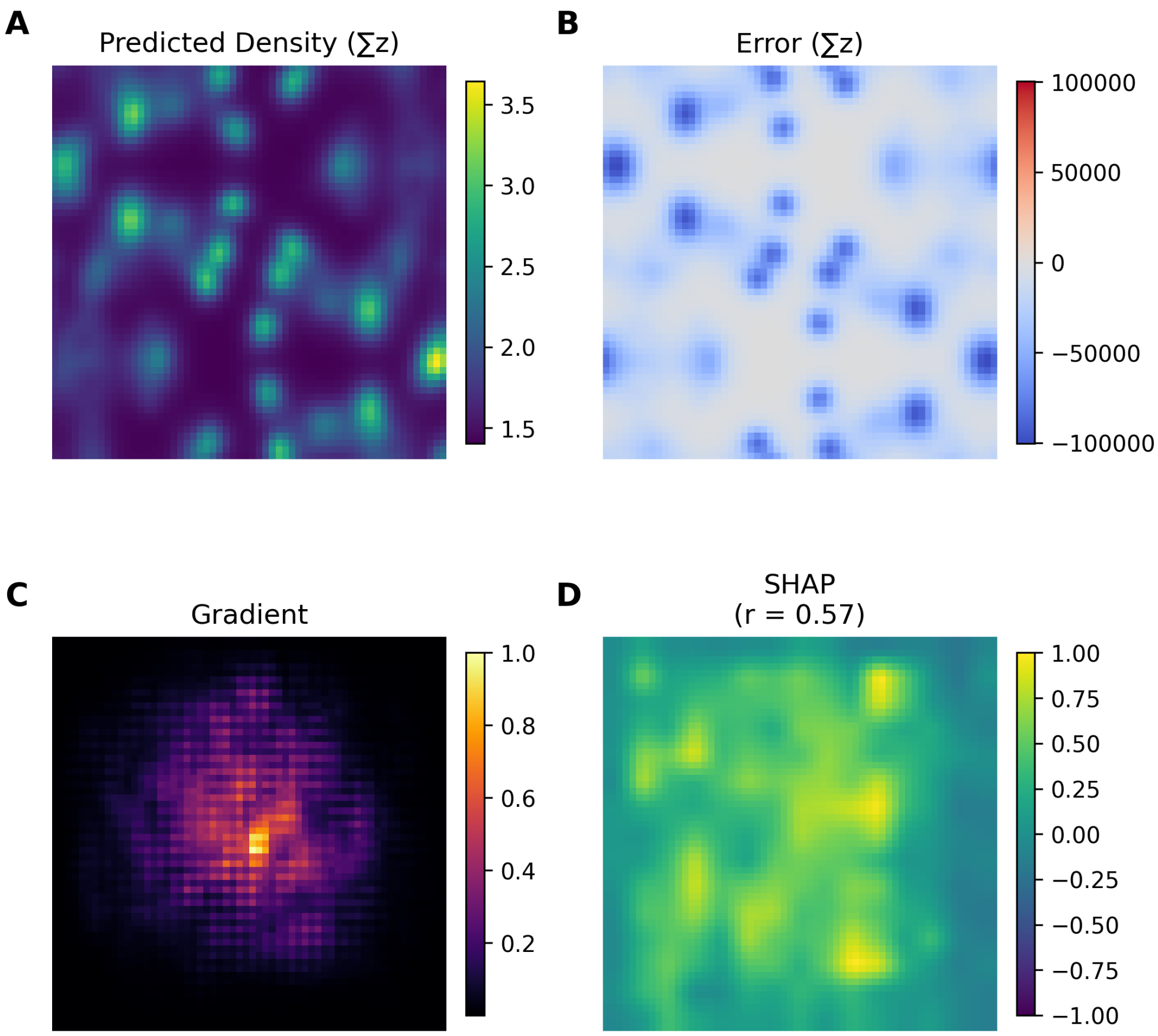

Figure S3: High Correlation Case ($r = 0.57$). Example where gradient and SHAP methods show moderate alignment. (A) Predicted charge density, (B) prediction error, (C) localized gradient saliency, and (D) SHAP attribution showing consistent spatial relevance.

- **Decoupled Regime ($r \approx 0$):** Local perturbations do not correspond to global importance, indicating that the model relies on broader contextual patterns rather than local heuristics (Figure S4).

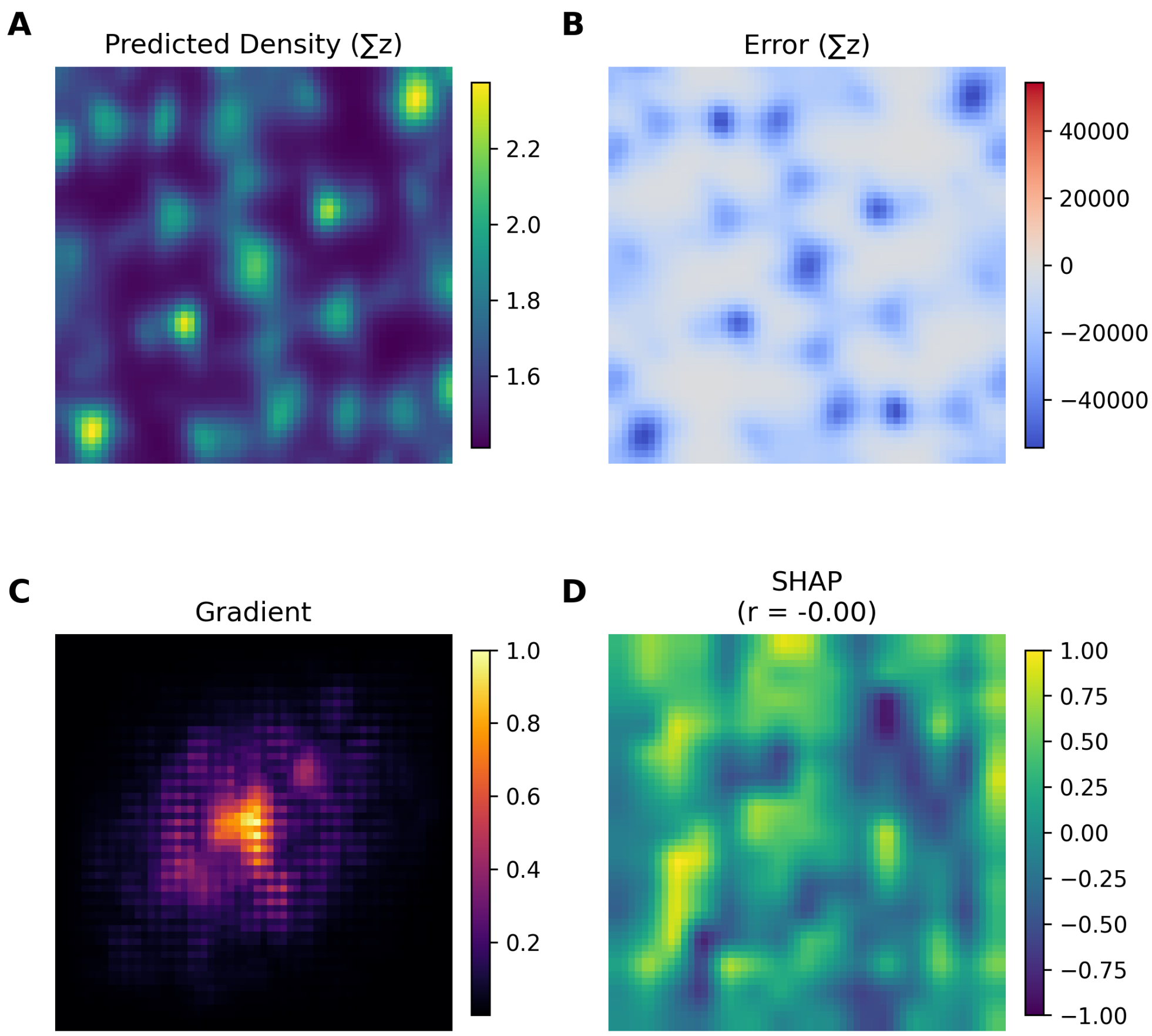

Figure S4: Low/Near-Zero Correlation Case ($r \approx -0.004$). Example of complete decoupling between attribution methods. While the gradient map (C) remains localized, the SHAP map (D) identifies entirely different distributed regions as globally important.

1.2.2 Role of Multi-channel Representation and Architecture

The 12-channel input representation is important for capturing this complexity. By encoding species-specific data and spatial descriptors into separate channels, the network introduces an inductive bias that encourages the model to process contributions separately before hierarchical integration.

From an explainability standpoint, gradient saliency highlights sensitivity within specific channels (local), whereas SHAP reflects how channel combinations contribute to the final prediction (global). The 3D U-Net architecture, through its encoder–decoder structure and skip connections, integrates these multi-scale features. This synergy provides a plausible

explanation for the coexistence of localized gradient responses and distributed SHAP attributions, suggesting that the model captures distributed structural correlations associated with the density field, beyond purely local peak matching.

2 Feature Selection and Channel Optimization

To determine the optimal input representation, a series of preliminary experiments were conducted using a "toy model" framework on the Materials Project (MP) database. The optimization process was divided into four distinct stages, focusing on the evolution of input descriptors and grid resolution.

The Baseline (Stage 1) utilized a single-channel input representing fundamental atomic properties, specifically atomic radius and mass. In Stage 2 (Intermediate), the input was expanded to 9 channels using a broader set of physical descriptors to capture more diverse chemical environments. Stage 3 (Channel Expansion) introduced the selected 12-channel feature set (described in the manuscript), which stabilized predictions even at a low resolution. Finally, Stage 4 (Final Production) transitioned the 12-channel set to the full 60^3 resolution grid (this result is presented in the OOS section of the MP database).

The quantitative results of this trajectory are summarized in Table S2 and in Figure S5.

Table S2: Performance trajectory across feature optimization and grid resolution stages. Metrics computed over the Materials Project dataset ($N = 308$).

Stage	Resolution	Mean Rel. Error (%)	Standard Error (SE)
Baseline (1 Channel)	20^3	19.17 ± 9.04	0.51
Intermediate (9 Channels)	20^3	17.31 ± 8.45	0.48
Channel Expansion (12 Ch)	20^3	11.25 ± 2.84	0.16
Final Production (12 Ch)	60^3	9.78 ± 6.74	0.38

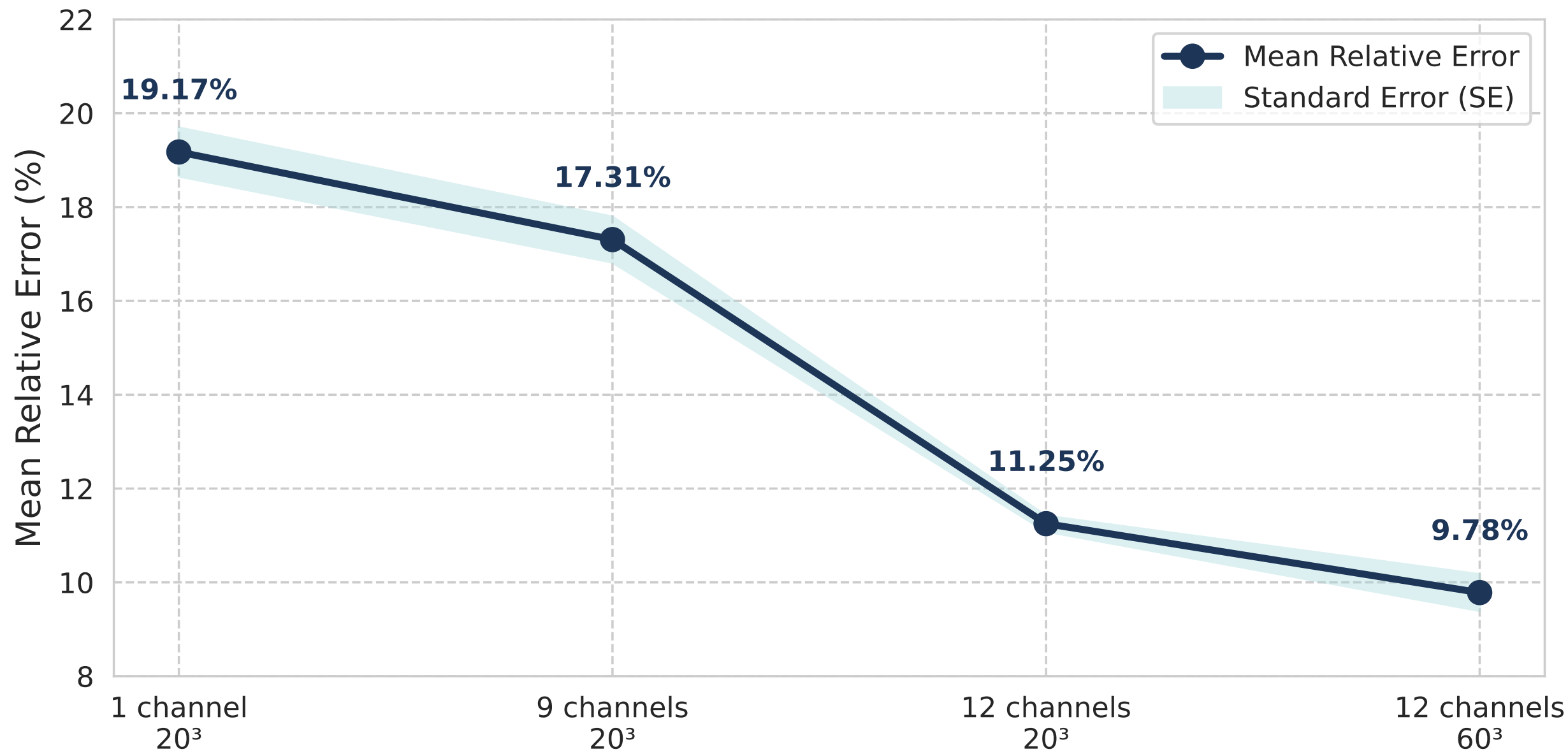

Figure S5: Model performance trajectory. The solid line indicates the Mean Relative Error, while the shaded region represents the Standard Error (SE) listed in Table S2. A marked improvement is observed upon adopting the 12-channel feature set.

The transition from a single-channel baseline to the 12-channel optimized input provided a total error reduction of approximately 49%. These results show that while raw physical properties provide a functional baseline, a refined multi-channel input is beneficial for capturing relevant nonlinear correlations in the electronic density across the tested crystal structures.

As shown in Table S2, the increase in standard deviation and SE at full resolution (60^3) compared to the toy resolution (20^3) for the 12-channel set is attributed to the model's exposure to finer spatial details and local density gradients. This represents a necessary trade-off for capturing high-frequency features and improving local structural fidelity.

2.1 Analysis of the 12-Channel Representational Advantage

The significant leap in performance observed when moving from 9 to 12 channels is consistent with improved representational capacity relative to the reduced-channel baselines tested here. While 9 channels provide a broader description than the baseline, they may lack

sufficient depth to isolate subtle correlations between specific atomic species and their local coordination environments.

The improved performance is consistent with a richer separation of element-level descriptors in the early convolutional layers of the 3D U-Net, which can act as an inductive bias before those features are integrated through the hierarchical architecture. The drop in both standard deviation and SE during the toy-resolution phase supports that this configuration improves predictive stability on the structurally heterogeneous carbon-containing subset derived from the Materials Project database.

3 Comparison with Existing Charge-Density Prediction Frameworks

To contextualize the development of Struct2ML within the state of the art, we contrast its design choices with representative machine-learning architectures for electronic charge-density estimation. These include the early molecular density-learning framework,[1] the local-environment Graph Convolutional Network (GCN),[2] the neural message-passing architecture DeepDFT[3] along with its subsequent equivariant extensions,[4] the density voxel approach,[5] and the higher-order equivariant ChargE3Net architecture.[6]

Supplementary Table S3 provides an expanded literature-based comparison of these methods. The values and descriptions summarized there should be interpreted strictly as reported within each original study and are not directly comparable across methods because of fundamental differences in datasets, density representations, grid or query resolutions, system sizes, hardware, and evaluation protocols.

Rather than claiming absolute numerical superiority over frameworks optimized purely for spatial transferability or invariant properties, the positioning of Struct2ML is framed around a practical compromise between throughput, storage footprint, and spatial fidelity. Symmetry-aware, higher-order equivariant networks achieve outstanding accuracy by embedding rigorous physical constraints, yet they inherently rely on computationally demanding message-passing pipelines involving tensor products.[6] Depending heavily on implementation, grid or query point sampling reconstruction, system size, and hardware configurations, reported inference costs for graph-based frameworks often scale non-trivially.

In contrast, Struct2ML targets a complementary engineering focus by mapping raw atomic distributions directly to a fixed 3D volumetric grid. This choice bypasses dynamic graph reconstruction during inference, allowing high-throughput volumetric processing and low-latency execution. While restricted in its intrinsic rotational equivariance treatment, this practical advance provides a useful improvement for high-throughput streaming and in-

teractive electronic-structure surrogate applications, maintaining robust performance across chemically heterogeneous systems such as those derived from the Materials Project subset ($R^2 = 0.799$, Mean Relative Error = 9.782%).

Table S3: Expanded literature-based comparison of representative electron-density prediction methods. Reported metrics and runtimes are not directly comparable because the studies differ in datasets, system sizes, density representations, grid/query resolutions, hardware, and evaluation protocols.

**Method**	**Systems & Dataset**	**Input & Density Rep.**	**Symmetry & Inductive Bias**	**Reported Validation & Utility**	**Limitations for Direct Comparison**	**Reported Inference Scale & Hardware**
Sinitskiy & Pande[1]	Organic molecules / molecular datasets (QM9-like).	Learns density corrections or fields from approximate quantum data in a volumetric grid.	No explicit $E(3)$-equivariance claimed in the original configuration.	Accelerates density and energy-related evaluations in molecular settings.	Different molecular dataset, input formulation, and target definitions; not a direct crystalline benchmark.	Milliseconds to seconds regime (System size and hardware dependent).
Gong et al. GCN[2]	Crystalline materials.	Local-environment graph representation; charge density predicted at grid points.	Geometry-aware local-environment descriptor; not a full higher-order $E(3)$-equivariant model.	Predicts material charge distributions and discusses derived properties/scaling.	Distinct network architecture and evaluation protocol; metrics cannot be directly ranked without matching environments.	Seconds regime (Highly dependent on target grid point sampling density).
DeepDFT[3]	Molecules, solids, and liquids depending on set.	Graph model with atom and query-point vertices; density evaluated at query points.	Message-passing and spatial locality. Original framework does not implement full intrinsic equivariance.	Accuracy-oriented charge-density prediction with graph representations.	Query-point coordinates and sampling density differ from fixed volumetric voxel representations.	Seconds to minutes range (Scales with the number of evaluation query points).
Equivariant GNN[4]	Molecules, liquids, and solids.	Equivariant graph neural network for fast electron-density estimation.	Explicit equivariant treatment embedded via spherical harmonics.	Demonstrates the physical benefit of symmetry-aware graph modeling for density fields.	Methodologically distinct from the original message-passing baseline framework.	Sub-second to seconds range (Dependent on spherical harmonics expansion order).
Del Rio et al.[5]	Materials Project subset.	Density voxel representation.	Local atomic environments mapped to structural grid fields.	Emulates density functional theory components (Density RMSE reported).	Independent validation sets, localized grid resolutions, and target coordinate scaling conventions.	Seconds to minutes range (Highly dependent on grid voxel resolution).
ChargE3Net[6]	Large materials dataset (Materials Project derived).	Higher-order $E(3)$-equivariant graph network for charge-density reconstruction.	Explicit $E(3)$-equivariant features and symmetry-constrained architecture.	State-of-the-art accuracy; demonstrates utility for accelerating self-consistent field (SCF) convergence.	Highly dependent on specific subset slicing, metric definitions, and target normalization protocols.	$\sim 10^2$ ms to seconds range (Dependent on higher-order tensor product operations).
**Struct2ML (This work)**	CH_4 AIMD, CO_2 out-of-domain tests, and carbon-bearing Materials Project subset.	Fixed 60^3 voxel grid; 12-channel atomic descriptor inputs; target density resampled to match grid.	Explicit periodic boundary conditions via modulo mapping; approximate translational equivariance.	Voxel-wise metrics, spatial correlation maps, out-of-domain testing, and ablation studies.	Target dataset restricted relative to the full materials space; downstream electronic-property validation remains limited.	**1 – 10 ms** (Fixed-grid inference on standard modern GPU hardware).

4 Training Dynamics and Convergence Profiles

To evaluate the numerical stability and optimization profile of the *Struct2ML* pipeline, the evolution of the multi-objective Combined Loss (integrating MAE, RMSE, gradient smoothness, and soft-Dice overlap) was monitored alongside the learning rate schedules across all three benchmark domains. Figures S6, S7, and S8 illustrate the training and validation histories for the Materials Project (MP) crystalline structures, the CH_4 molecular snapshots, and the CO_2 molecular snapshots, respectively.

Across all datasets, the optimization profiles exhibit a consistent two-phase convergence regime. The initial phase (the first 100–200 epochs) is characterized by a steep, monotonic decline in both training and validation errors, representing the rapid acquisition of large-scale spatial structures and global charge envelopes. The second phase consists of a long-horizon refinement plateau where localized electronic features and peak boundaries are fine-tuned.

- **Materials Project Crystals (Figure S6):** Optimized over 2,000 epochs, the loss trajectory demonstrates exceptional stability under the complex multi-component loss weightings, with no signs of gradient divergence despite the structural and compositional diversity of the 6,355 crystalline systems.

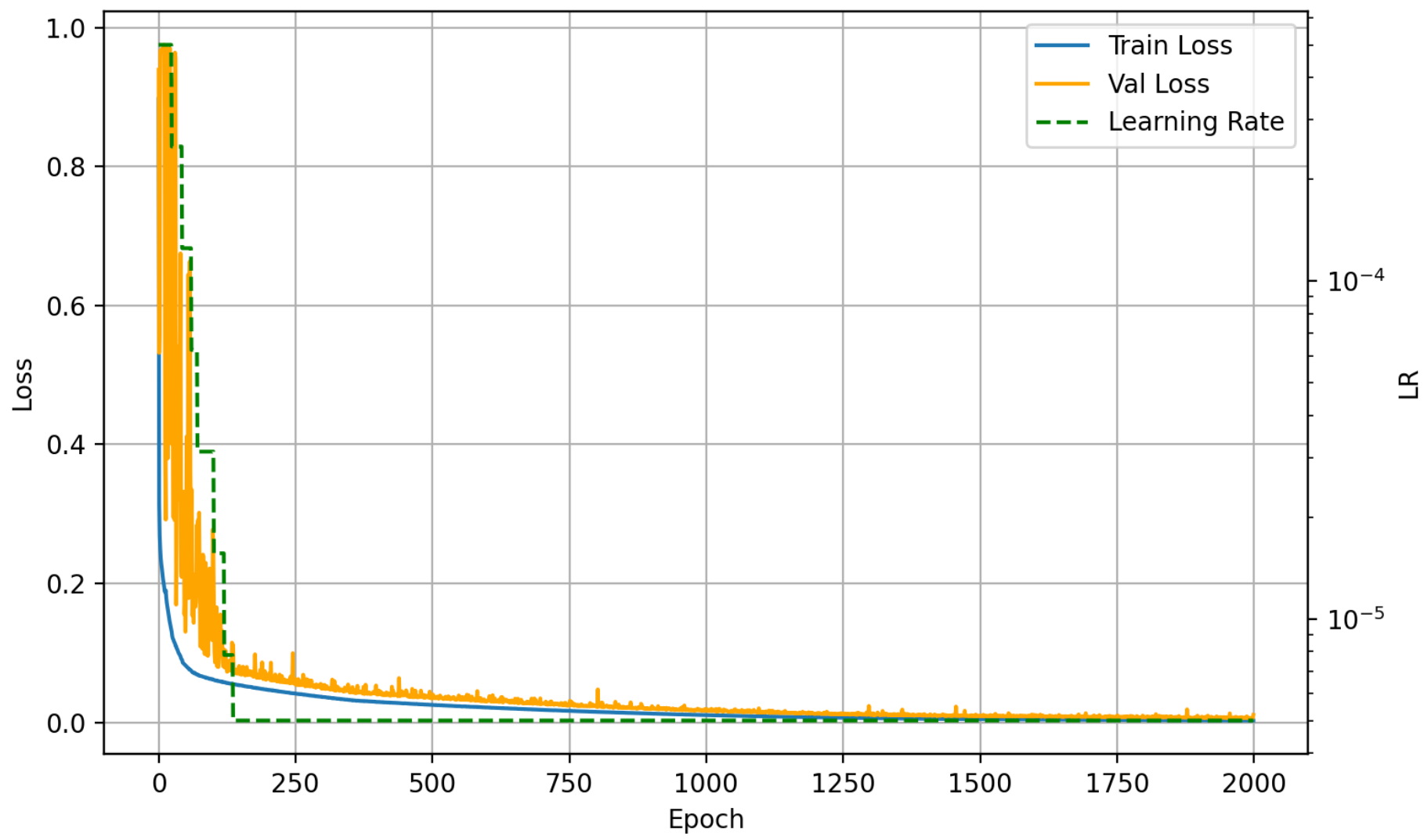

Figure S6: Materials Project convergence profile over 2,000 epochs. The loss trajectory demonstrates high stability under complex multi-component loss weightings across the 6,355 crystalline systems.

- **CH_4 Molecular Trajectories (Figure S7):** Progressing over 1,500 epochs, convergence remains highly stable. The tight tracking between the training and validation curves confirms that the model generalizes robustly to dynamic structural distortions without overfitting the localized atomic displacements.

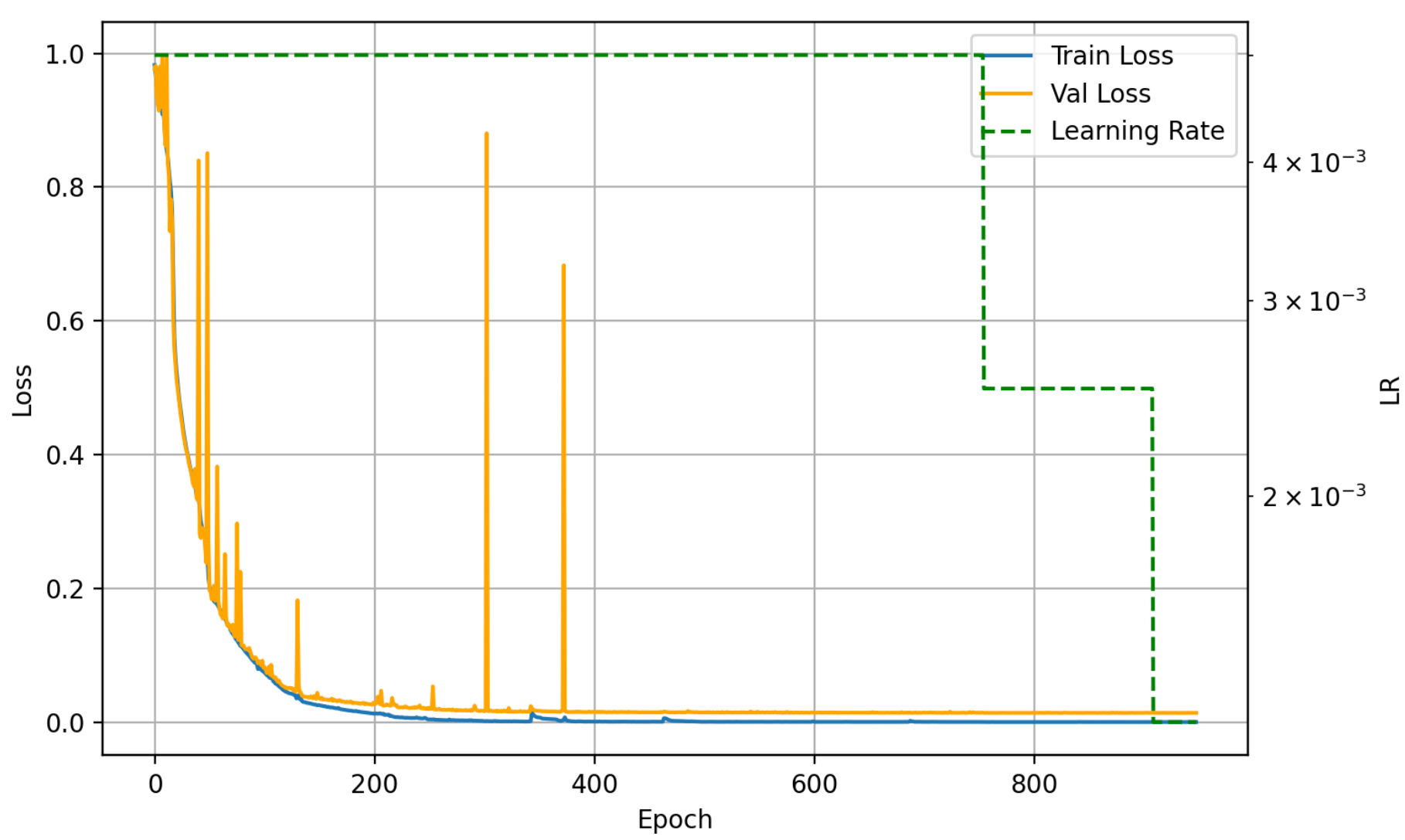

Figure S7: CH_4 molecular dataset training dynamics over 1,500 epochs. The tight tracking between curves confirms generalization to dynamic structural distortions without overfitting.

- **CO_2 Molecular Trajectories (Figure S8):** The convergence profile demonstrates the utility of the learning-rate schedule. Following an initial plateau, the step-decay applied after epoch 800 efficiently minimizes residual high-frequency voxel errors, accelerating final convergence.

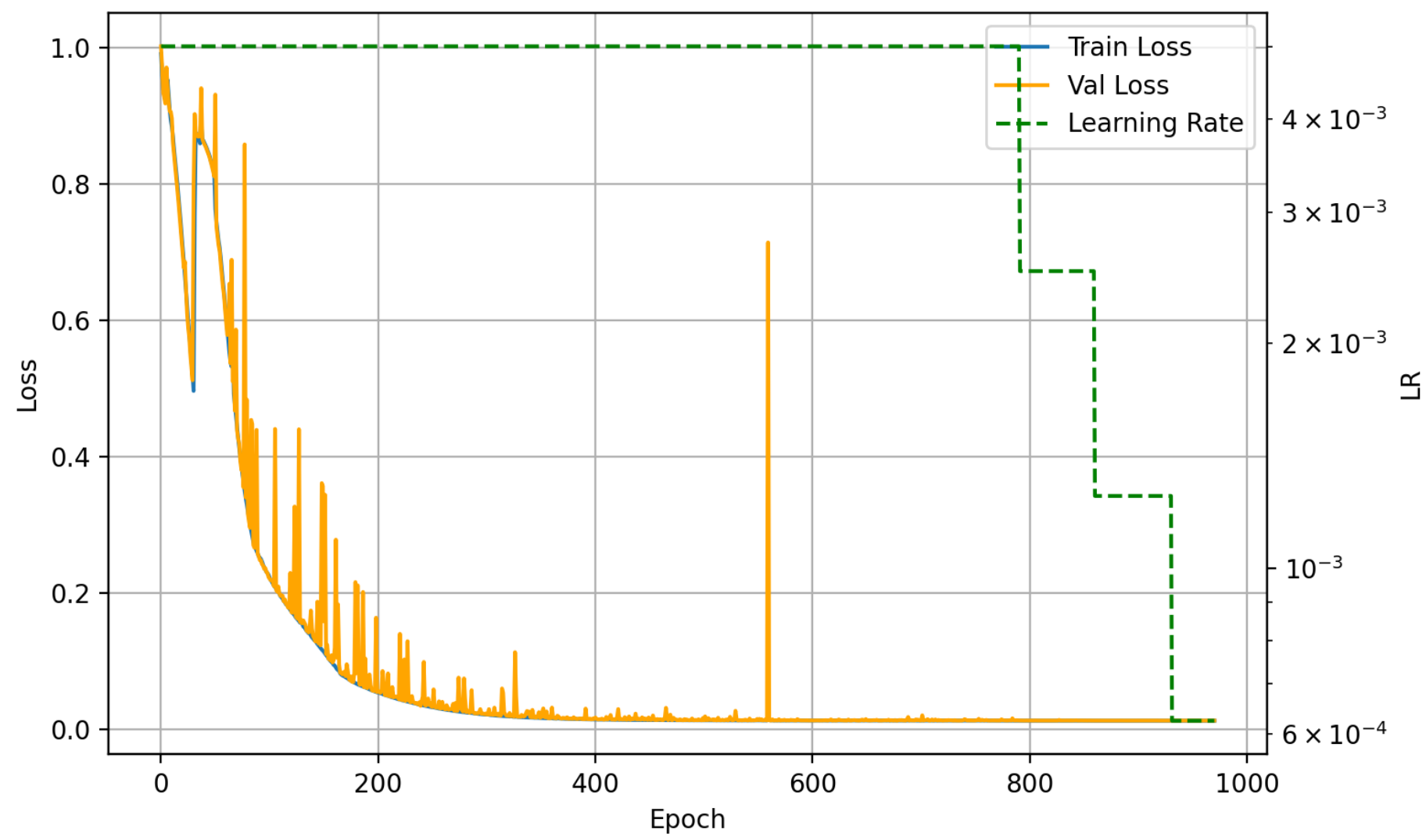

Figure S8: CO_2 dataset convergence profile. The step-decay applied after epoch 800 efficiently minimizes residual high-frequency voxel errors, accelerating final convergence.

In all cases, the systematic absence of a divergence gap between the training and validation trajectories demonstrates the regularization benefits of embedding physically motivated atomic descriptors onto a fixed periodic grid, ensuring reliable convergence regardless of the target chemical domain.

References

(1) Sinitskiy, A. V.; Pande, V. S. Deep Neural Network Computes Electron Densities and Energies of a Large Set of Organic Molecules Faster than Density Functional Theory (DFT). *arXiv preprint arXiv:1809.02723* **2018**,

(2) Gong, S.; Xie, T.; Zhu, T.; Wang, S.; Fadel, E. R.; Li, Y.; Grossman, J. C. Predicting charge density distribution of materials using a local-environment-based graph convolutional network. *Physical Review B* **2019**, *100*, 184103.

(3) Jørgensen, P. B.; Bhowmik, A. DeepDFT: Neural Message Passing Network for Accurate Charge Density Prediction. NeurIPS 2021 Workshop on Machine Learning and the Physical Sciences. 2021; arXiv preprint arXiv:2011.03346.

(4) Jørgensen, P. B.; Bhowmik, A. Equivariant graph neural networks for fast electron density estimation of molecules, liquids, and solids. *npj Computational Materials* **2022**, *8*, 183.

(5) del Rio, B. G.; Phan, B.; Ramprasad, R. A deep learning framework to emulate density functional theory. *npj Computational Materials* **2023**, *9*, 158.

(6) Koker, T.; Quigley, K.; Taw, E.; Tibbetts, K.; Li, L. Higher-order equivariant neural networks for charge density prediction in materials. *npj Computational Materials* **2024**, *10*, 161.
